# Evolution of translational control and the emergence of genes and open reading frames in human and non-human primate hearts

**DOI:** 10.1038/s44161-024-00544-7

**Published:** 2024-09-24

**Authors:** Jorge Ruiz-Orera, Duncan C. Miller, Johannes Greiner, Carolin Genehr, Aliki Grammatikaki, Susanne Blachut, Jeanne Mbebi, Giannino Patone, Anna Myronova, Eleonora Adami, Nikita Dewani, Ning Liang, Oliver Hummel, Michael B. Muecke, Thomas B. Hildebrandt, Guido Fritsch, Lisa Schrade, Wolfram H. Zimmermann, Ivanela Kondova, Sebastian Diecke, Sebastiaan van Heesch, Norbert Hübner

**Affiliations:** 1 Cardiovascular and Metabolic Sciences, Max Delbrück Center for Molecular Medicine in the Helmholtz Association (MDC), Berlin, Germany; 2 Max-Delbrück-Center for Molecular Medicine in the Helmholtz Association (MDC), Technology Platform Pluripotent Stem Cells, Berlin, Germany; 3 Leibniz Institute for Zoo and Wildlife Research, Berlin, Germany; 4 Freie Universitaet Berlin, Berlin, Germany; 5 Institute of Pharmacology and Toxicology, University Medical Center Göttingen, Göttingen, Germany; 6 DZHK (German Center for Cardiovascular Research), Partner Site Lower Saxony, Göttingen, Germany; 7 DZNE (German Center for Neurodegenerative Diseases), Göttingen, Germany; 8 Fraunhofer Institute for Translational Medicine and Pharmacology (ITMP), Göttingen, Germany; 9 Biomedical Primate Research Centre (BPRC), Rijswijk, The Netherlands; 10 DZHK (German Center for Cardiovascular Research), Partner Site Berlin, Berlin, Germany; 11 Princess Máxima Center for Pediatric Oncology, Utrecht, The Netherlands; 12 Oncode Institute, Utrecht, The Netherlands; 13 Charité-Universitätsmedizin, Berlin, Germany; 14 Helmholtz Institute for Translational AngioCardioScience (HI-TAC) of the Max Delbrück Center for Molecular Medicine in the Helmholtz Association (MDC) at Heidelberg University, Heidelberg, Germany

**Keywords:** Cardiovascular genetics, Transcriptomics, Heart development, Phylogenomics

## Abstract

Evolutionary innovations can be driven by changes in the rates of RNA translation and the emergence of new genes and small open reading frames (sORFs). In this study, we characterized the transcriptional and translational landscape of the hearts of four primate and two rodent species through integrative ribosome and transcriptomic profiling, including adult left ventricle tissues and induced pluripotent stem cell-derived cardiomyocyte cell cultures. We show here that the translational efficiencies of subunits of the mitochondrial oxidative phosphorylation chain complexes IV and V evolved rapidly across mammalian evolution. Moreover, we discovered hundreds of species-specific and lineage-specific genomic innovations that emerged during primate evolution in the heart, including 551 genes, 504 sORFs and 76 evolutionarily conserved genes displaying human-specific cardiac-enriched expression. Overall, our work describes the evolutionary processes and mechanisms that have shaped cardiac transcription and translation in recent primate evolution and sheds light on how these can contribute to cardiac development and disease.

## Abstract (web summary)

Ruiz-Orera et al. used comparative transcriptomics and translatomics to analyze the cardiac evolution in primates and discovered species-specific and lineage-specific genomic innovations that might contribute to cardiac development and disease.

## Main

Human and non-human primate (NHP) genomes share considerable genetic similarity, yet their organs, including the heart, present numerous anatomical and molecular differences. This also holds true for primate hearts, which exhibit differences in rate, cardiac output and left ventricular morphology^1,2^. Furthermore, primates vary in their susceptibility to certain types of cardiovascular disease, such as atherosclerosis and myocardial fibrosis^3,4^. Understanding these differences in cardiac physiology, development and disease susceptibility at the molecular level is crucial for advancing knowledge of primate biology and evolution.

Previous studies demonstrated the importance of primate transcriptomic regulation and diversity in function, development, sex and evolution^5–11^. However, the impact of translational control on the heart in an evolutionary context remains unexplored. In human hearts, the translational control of mRNAs plays a critical role in cardiac failure, fibrosis and vascular dysfunction^12–15^. For example, the muscle-specific ribosomal protein RPL3L influences translation elongation of cardiac contraction genes in adult hearts^16^ while also modulating mitochondrial activity^17^. How this prominent role for translational regulation evolved, and which differences exist between primates across cardiac development and function, is still unknown.

Evolutionarily young genes and short open reading frames (sORFs) comprise the fastest and most dynamically evolving class of putative functional factors in primate genomes. Thousands of newly evolved genes and sORFs that might serve regulatory roles and/or encode microproteins were recently discovered in various human cell types and tissues^15,18–21^ and can emerge de novo from ancestral intergenic regions^21–23^. This dramatic putative expansion of the human transcriptome and translatome has increased the complexity of factors to consider when investigating cardiac function^15^. Whether young genes and sORFs could contribute to human and primate uniqueness in cardiac development and disease is not known.

In the present study, we examined gene transcription, translation and sORF emergence in human and NHP hearts. We profiled the transcriptomes and translatomes of 24 adult left ventricles (LVs) from humans, chimpanzees and rhesus and 14 induced pluripotent stem cell-derived cardiomyocyte (iPSC-CM) cultures from humans, chimpanzees, gorillas and rhesus. This extensive dataset represents a powerful resource for interrogating regulatory differences between humans and NHPs across developmental and evolutionary stages. Additionally, we included 11 LVs from mice and rats as evolutionary outgroups. We identified evolutionarily young and conserved regulators of cardiac function through mRNA abundance, ribosome occupancy and translational efficiency. We describe here the evolutionary innovations that shaped the cardiac transcriptomes and translatomes, identifying 551 and 504 evolutionarily young genes and sORFs, respectively. We found 76 human genes with recently evolved cardiac-enriched expression, such as *CMAHP*, a hydroxilase involved in sialic acid metabolism that was pseudogenized 2–3 million years ago (Mya)^24^, and an Alu-derived isoform encoded by the sodium-glucose co-transporter gene *SGLT1*. We show that evolutionarily young genes and sORFs can be biologically active and show dysregulation in heart failure. Our study offers insights into genetic and molecular mechanisms affecting cardiac function, development and disease, showcasing the differences among primate species.

## Results

### Transcriptomes and translatomes of adult mammalian hearts

**Fig. 1:**
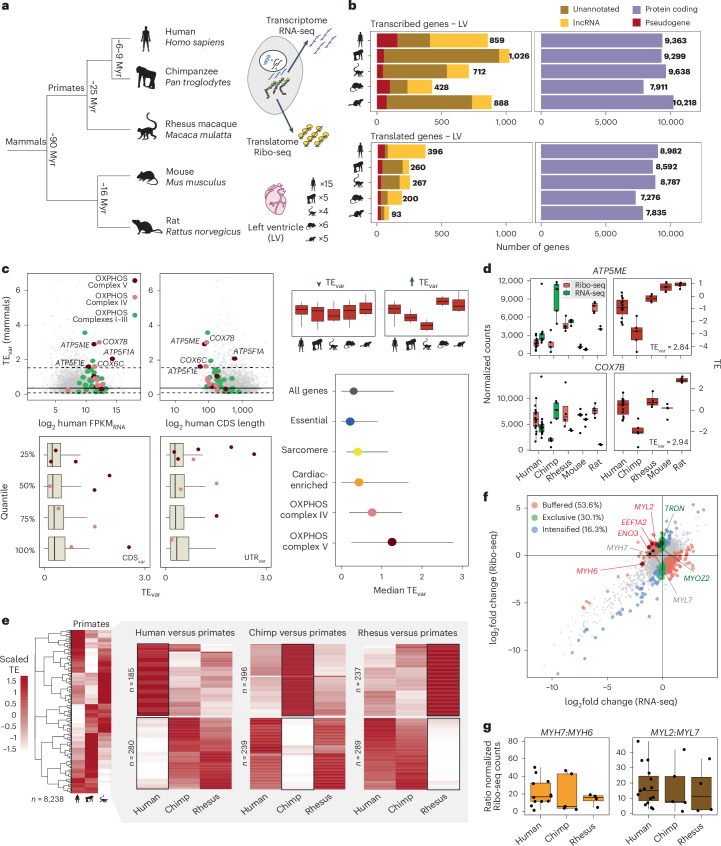
Translational control across primate and mammalian adult hearts. **a**, Estimated evolutionary distances and schematic of the biological LV replicates (*n*_human_ = 15, *n*_chimp_ = 5, *n*_rhesus_ = 4, *n*_mouse_ = 6 and *n*_rat_ = 5). **b**, Number of transcribed and translated genes in LVs, divided by species and gene biotype. **c**, Top, distribution of TE_var_ compared to the levels of RNA-seq expression (log_2_ FPKM) and CDS length of each gene across human LVs. The right top box plot represents two hypothetical genes with low and high TE_var_. Horizontal lines represent the average (non-dashed) and 5th and 95th percentiled (dashed) of TE_var_. Specific gene groups based on different OXPHOS complexes are highlighted. Left bottom, box plots with TE_var_ for four quantile CDS groups based on the inter-species variance in CDS (CDS_var_) or UTR length (UTR_var_). Differences in TE_var_ across quantiles were significant for CDS_var_ (ANOVA, *P* = 1.3 × 10^−7^) and UTR_var_ (ANOVA, *P* = 6.9 × 10^−4^). Right bottom, dot plot with median TE variances for specific gene groups. Horizontal lines represent 95% CIs. **d**, Box plots with the distribution of normalized RNA-seq counts, Ribo-seq counts and TE for two genes with high TE_var_. **e**, Heatmaps with scaled TE of 8,238 genes in the three primate species, including subsets of genes with species-specific TE changes. **f**, Differential RNA-seq and Ribo-seq expression between human and NHPs. Genes with human-specific regulation of translation (*n* = 465) are depicted in red, green or blue. Of these, 53.6% of genes were differentially expressed at the RNA-seq level and TE in opposite directions (buffering); 30.1% of the genes showed specific regulation at the Ribo-seq level (exclusive); and 16.3% of the genes displayed translational regulation that intensified the changes in transcript abundance (intensified). *MYH7*, *MYL7* and six cardiac-enriched genes with human-specific TE changes are highlighted. **g**, Box plots with *MYH7:MYH6* and *MYL2:MYL7* ratios based on normalized Ribo-seq counts across primate LVs. In **c**, **d** and **g**, all biological replicates described in **a** were included, with boxes indicating interquartile range (IQR; 25th and 75th percentiles), center line indicating median and whiskers indicating minimum to maximum. Myr, million years. Source data

To explore the evolution of translational control in primate and mammalian hearts, we generated ribosome sequencing (Ribo-seq) and RNA sequencing (RNA-seq) data from the adult LVs of five chimpanzees and four rhesus macaques (Fig. 1a and Supplementary Table 1). We additionally included data from 15 human adult LVs that were previously generated in our laboratory^15^. These three species represent the catarrhini lineage (~25 Mya) within the primate taxa (Fig. 1a). As non-primate outgroup species, we added 11 LV datasets from mouse and rat.

**Extended Data Fig. 1:**
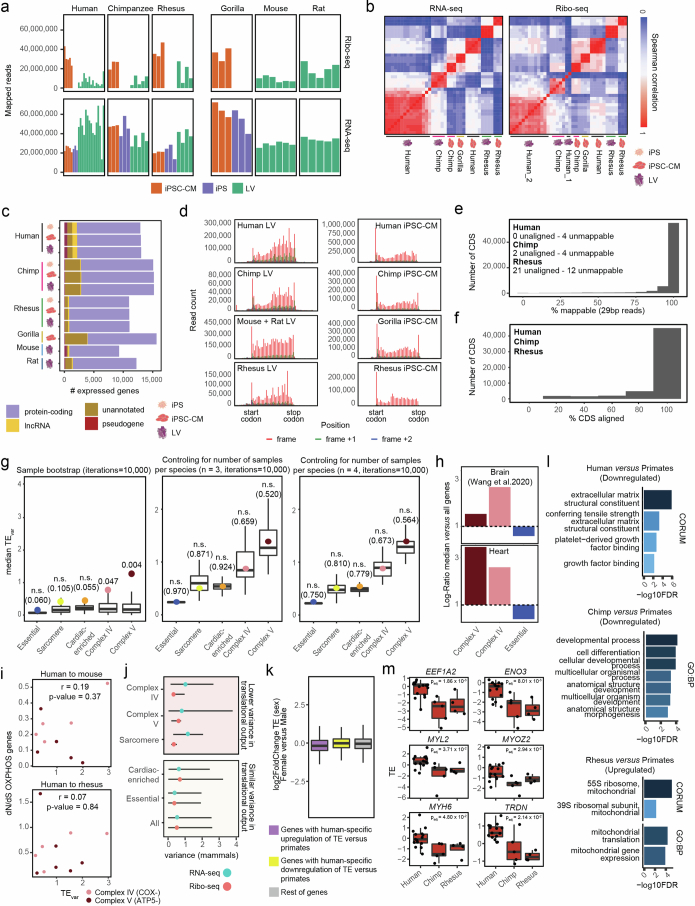
Transcription and translation across adult hearts and iPSC-CMs. **a)** Bar plots indicating the number of mapped RNA-seq and Ribo-seq reads per sample. **b)** Spearman correlations of gene FPKM values across RNA-seq and Ribo-seq samples. Only defined robust orthologous genes across all species were considered. **c)** Total number of assembled genes in pooled datasets of iPS cells, iPSC-CMs, and adult heart left ventricles (LV), divided by species and gene biotype. **d)** Distribution of Ribo-seq read periodicities between annotated start and stop codons for pooled iPSC-CM and LV samples in human, chimpanzee, gorilla, rhesus, and rodents (mouse and rat). The correct frame is displayed in red. **e)** Histogram depicting the number of human, chimp, and rhesus CDS sequences mappable by reads of 29 base pairs (the average size of ribosome-protected fragments) across the other primate genomes considered in the study. The accompanying text describes the count of CDS sequences that remain unmappable or unaligned to any other primate genome. **f)** Histogram illustrating the difference in length of CDS sequences shared by the identified homologous CDS across other primate genomes. **g)** Box plots displaying the median TE variances for each defined gene group following sample resampling (left, 10,000 iterations) or data downsampling (10,000 iterations with three samples per species, and 10,000 iterations with four samples species). In the left plot, adjusted FDR p-values represent if observed TE variation is higher than in resampled samples. In the middle and right plots, adjusted FDR p-values are calculated to evaluate if the observed TE variation is statistically different to the distribution of TE variances with an altered number of samples. Please see **Methods** for further information about the used statistical tests. **h)** Logarithmic ratio of median TE variances from genes from OXPHOS complexes V and IV, and essential genes, divided by all expressed genes in brain and heart. Brain data was retrieved from Wang et al. ^26^. Only human, rhesus, and mouse data was used to calculate TE variances. **i)** Pearson’s correlation of variances in TE and ratios of non-synonymous to synonymous substitutions (dN/dS) between human and mouse and human and rhesus for nuclear components of the Complex IV and V OXPHOS pathway. **j)** Dot plot with median mRNA abundance (RNA-seq) and ribosome occupancy (Ribo-seq) variances for specific gene groups. Boxes are grouped by species, representing biological LV replicates (n_human_ = 15, n_chimp_ = 5, n_rhesus_ = 4, n_mouse_ = 6, n_rat_ = 5). Horizontal lines represent 95% confidence intervals. **k)** Box plots with logarithmic fold changes of TE between female (n = 7) and male (n = 8) human samples. None of the 465 genes whose TE were significantly up- or down-regulated in humans showed a significant difference in TE between both sexes. **l)** CORUM pathways and GO terms significantly enriched in the group of genes with significant differences in TE. Other comparisons did not result in enriched functions. **m)** Box plots with the distribution of TE values of six cardiac-enriched genes with significantly increased TE in humans *versus* non-human primates. Adjusted p-values are calculated by DESeq2. Boxes are grouped by species, representing biological LV replicates (n_human_ = 15, n_chimp_ = 5, n_rhesus_ = 4). In panels **g)**, **k)**, and **m)**, boxes indicate interquartile range (IQR; 25th and 75th percentiles); center line indicates median (50th percentile); whiskers indicate minimum to maximum. Source data

Annotation of NHP transcriptomes is limited and primarily based on homology to human annotations^25^. Here, we mapped the RNA-seq datasets to the corresponding species genomes, refined existing annotations and generated assembled transcriptomes, resulting in an average of 12,164 genes per species (Extended Data Fig. 1a–c), of which 10,068 genes were actively transcribed in at least three samples and were used to define adult cardiac transcriptomes (Fig. 1b). Next, we mapped the Ribo-seq data to the assembled transcriptomes, yielding an average of 38.1 million mapped reads per library, with a high correlation across biological replicates (Extended Data Fig. 1b). The Ribo-seq data showed high quality, with approximately 82–84% of the 29-nucleotide-long ribosome-protected fragments mapping to the primary reading frame of annotated coding sequences (CDSs) (Extended Data Fig. 1d). We identified an average of 8,538 translated mammalian genes with at least one CDS displaying significant three-nucleotide codon-moving periodicity, including a total of 611 genes annotated as long non-coding RNAs (lncRNAs), 174 pseudogenes and 431 unannotated genes (Fig. 1b and Methods).

### Evolution of translational control across mammalian hearts

Evolution of regulatory processes can selectively control transcriptome and translatome expression as previously shown for the mammalian brain, liver and testis^26^. Here, we sought to determine whether evolution also shaped the translational regulation of specific gene groups across mammalian hearts. To this end, we calculated translational efficiencies (TEs) by computing the ratio of normalized Ribo-seq and RNA-seq counts per CDS.

Next, we evaluated the preservation of TE over mammalian evolution by calculating variance in TE across species (TE_var_) for a subset of 4,524 translated genes with robust mammalian orthologs (Extended Data Fig. 1e–gand Methods). Genes with specific cardiac functions, such as sarcomeric genes and genes with previously defined enriched expression in heart and muscle tissue^15^, displayed average variance in TE (median TE_var_ of 0.40–0.43; Fig. 1c and Extended Data Fig. 1g). In contrast, genes previously defined as essential for cellular function^27^ exhibited limited divergence between transcription and translation levels, probably due to strong evolutionary constraints that preserve expression levels (median TE_var_ of 0.29; Fig. 1c). Of note, nuclear-encoded genes of mitochondrial oxidative phosphorylation (OXPHOS) complexes IV (cytochrome c oxidase) and V (ATP synthase) displayed the highest TE variation across the investigated mammalian hearts (median TE_var_ of 0.76 and 1.26, respectively; Fig. 1c). Resampling analyses indicated that the observed high TE_var_ of OXPHOS complex IV and V subunits reached significance (*P* = 0.047 and *P* = 0.004, respectively; Extended Data Fig. 1g and Methods). Among the genes with the highest variation in TE were *ATP5ME* (complex V) and *COX7B* (complex IV) (Fig. 1d). The observed high variance in OXPHOS complexes IV and V was not driven by the levels of gene expression, CDS length or variations in CDS and untraslated region (UTR) length across species (Fig. 1c). For comparison, we calculated gene variance in TE for a public dataset of mammalian brain tissues^26^ and observed similarly high variation for complex IV but not for complex V (Extended Data Fig. 1h). Although components of the complex V are known to evolve rapidly across primate evolution^28^, the TE_var_ levels of nuclear-encoded components of the complex V did not correlate with the strength of purifying selection acting on their protein-coding sequences (Extended Data Fig. 1i). Therefore, the cardiac divergence of translational control in this specific OXPHOS complex is likely not driven by the faster evolution of their protein sequences. For both complexes IV and V, the variance at the transcriptional level exceeded that at the translational level (Extended Data Fig. 1j), indicating that the observed high TE_var_ may compensate for increased transcriptional variation by counterbalancing levels of translational abundance.

In summary, our results demonstrate that mammalian evolution was shaped by changes in the regulation of cardiac translatomes, with mitochondrial OXPHOS complexes IV and V among the most impacted by TE changes.

### Species-specific translational control of primate cardiac genes

To characterize the evolution of cardiac translational control during recent human, chimpanzee and rhesus evolution, we compiled a list of genes whose TEs were significantly upregulated or downregulated in each specific species, as compared to the other two primate species (*n*_human_ = 465, *n*_chimp_ = 635, *n*_rhesus_ = 526; Fig. 1e). These species-specific changes in TE were often driven by translational buffering (53.6% for human genes), which opposes the impact of alterations in mRNA levels (Fig. 1f). We next compared TE differences across sexes in genes with human-specific changes in TE, but the differences observed were minimal, with no significant changes identified among the list of 465 human genes (Extended Data Fig. 1k). Consequently, it is unlikely that the observed species-specific variations in TE are attributable to an imbalance in the distribution of sexes within the primate sample set.

In rhesus macaque, components of the mitoribosome were significantly upregulated (Extended Data Fig. 1l). Chimpanzee genes with recent species-specific downregulated TE changes were involved in developmental processes and morphogenesis. In contrast, negative changes in TE in humans were associated with structural components of growth factor binding and extracellular matrix genes. Of note, we previously found that the translation of extracellular matrix components was specifically regulated in the opposite direction in heart failure^15^.

We also noted that the TEs of six cardiac-enriched genes, including cardiac-specific translation factor *EEF1A2*, enolase *ENO3*, calcium release factor *TRDN* and the sarcomere proteins *MYH6*, *MYL2* and *MYOZ2*, were significantly higher in humans as compared to other primates (Extended Data Fig. 1m). *EEF1A2*, *ENO3*, *MYH6* and *MYL2* exhibited regulatory patterns consistent with translational buffering, compensating for the lower levels of transcriptional abundance observed in humans as compared to other primates. In contrast, *MYOZ2* and *TRDN* demonstrated an exclusive directionality of regulation in translational output (Fig. 1f). Notably, human-specific translational upregulation of *MYH6* and *MYL2* did not result in skewed ratios of translated *MYH7:MYH6* or *MYL2:MYL7* compared to other species (Fig. 1g). This may suggest a complex interplay of translational regulatory mechanisms given that the expression ratios of these marker pairs can be used to define the developmental stage or chamber of the primate heart^29^.

### Primate iPSC-CMs as a model of fetal hearts

**Fig. 2:**
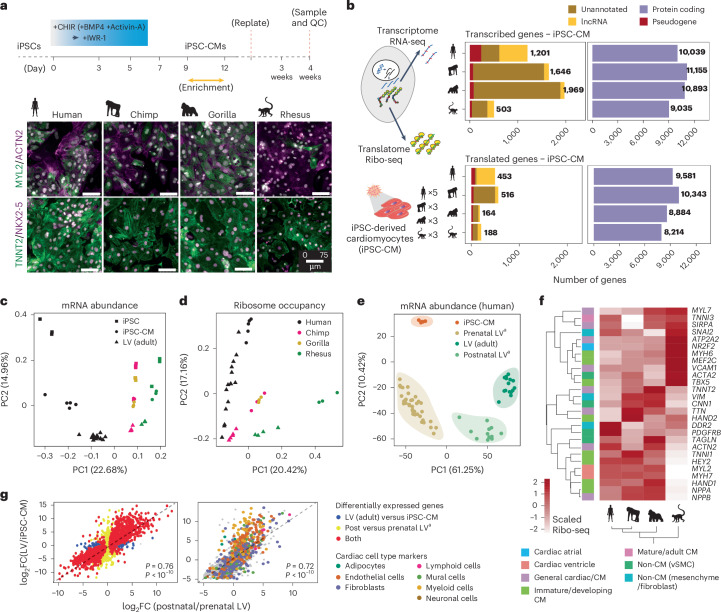
Primate iPSC-CMs are models of fetal cardiac development. **a**, Top, schematic of the iPSC differentiation protocol. BMP4 and Activin A were required only for rhesus iPSC differentiation (Methods). We generated five differentiation replicates for human and three differentiation replicates for chimpanzee, gorilla and rhesus. Bottom, immunocytochemistry of iPSC-CMs after 4 weeks of differentiation revealed expression of cardiac and CM markers. Merged images with 4′,6-diamidino-2-phenylindole (DAPI) counterstain (gray). Bar, 75 μm, being similar for all species. **b**, Left, schematic of the generated iPSC-CM samples, representing four primate species (human, chimpanzee, gorilla and rhesus macaque). Right, total number of transcribed and translated genes in iPSC-CMs, divided by species and gene biotype. Only genes transcribed in a minimum of three samples (FPKM ≥ 0.5) and an average FPKM ≥ 1 were selected. **c**,**d**, PCA of the mRNA abundance (**c**) and Ribo-seq occupancy (**d**) of 6,722 genes with robust mammalian orthologs across iPSCs, iPSC-CMs and adult LVs of the primate species. **e**, PCA of the mRNA abundance of iPSC-CMs, adult LVs and reanalyzed prenatal and postnatal LV data from Cardoso-Moreira et al.^9^. **f**, Heatmap with scaled Ribo-seq counts for selected markers in all generated primate iPSC-CMs. Only defined consistent orthologous genes across primate species were considered (Methods). **g**, Dot plots with the log_2_ fold changes of gene transcription between adult LVs and iPSC-CMs (*y* axis) and between matched postnatal and prenatal heart samples (*x* axis) in the dataset from Cardoso-Moreira et al.^9^. Cell-type-specific markers of the non-cardiomyocyte cardiac cell types are highlighted in different colors. Spearman’s rank correlation coefficients and one-sided *P* values are also displayed. FC, fold change; PC, principal component; QC, quality control; vSMC, vascular smooth muscle cell. ^a^Analysis performed using data from Cardoso-Moreira et al.^9^. Source data

**Extended Data Fig. 2:**
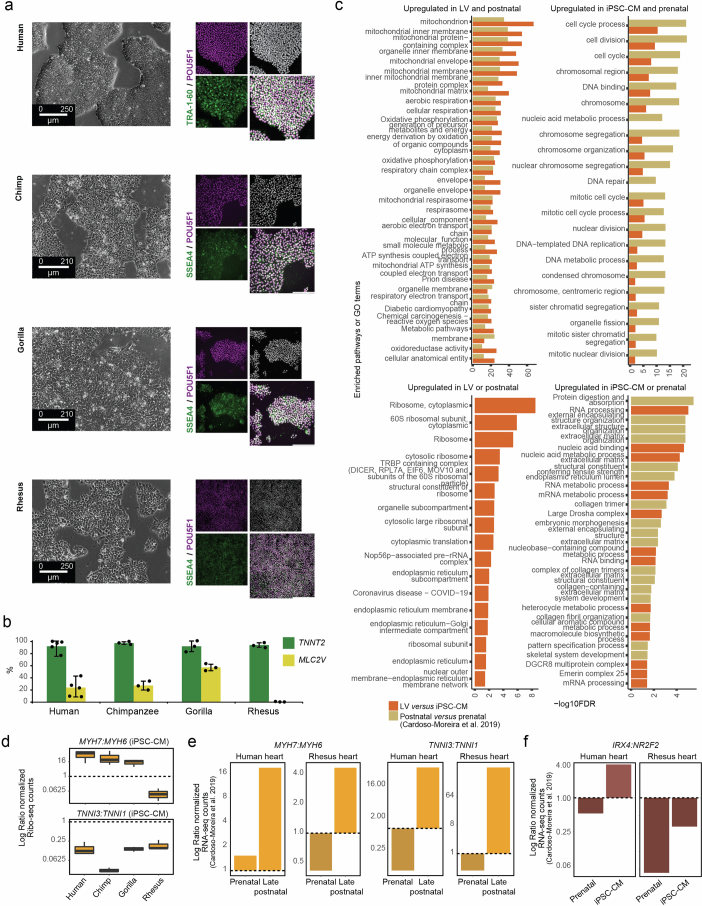
Validation of iPSC reprogramming and iPSC-CM differentiation. **a)** Microscopy of human and NHP iPSCs by live-cell phase contrast (left column) and fixed-cell immunofluorescence following immunostaining against pluripotency-associated markers (right column). Bar = 250 μm (human, rhesus) and 210 μm (chimp, gorilla). **b)** Mean quantification of CM marker expression across iPSC-CMs at 4 weeks of differentiation. We included five independent differentiation replicates for human and three differentiation replicates for chimp, gorilla, and rhesus, error bars ± S.D. are represented. **c)** GO terms enriched in human genes upregulated in adult LV or iPSC-CMs, and/or in the corresponding pre- and postnatal developmental cardiac stages from Cardoso-Moreira et al. ^9^. The observed differences led to enrichments in similar functional pathways in both datasets, mostly related to mitochondrial functions, OXPHOS, and cell cycle. In contrast, enriched functions that were uniquely regulated in one dataset included cytoplasmic translation, RNA processing, or extracellular matrix organization, among others. **d)** Box plots with *MYH7:MYH6* and *TNII3:TNNI1* ratios based on the number of normalized Ribo-seq counts across primate iPSC-CMs. Boxes indicate interquartile range (IQR; 25th and 75th percentiles); center line indicates median (50th percentile); whiskers indicate minimum to maximum. We included five differentiation replicates for human and three differentiation replicates for chimp, gorilla, and rhesus. **e)** *MYH7:MYH6* and *TNII3:TNNI1* ratios for human and rhesus hearts based on normalized RNA-seq counts from the three earliest matching prenatal and three oldest postnatal samples from Cardoso-Moreira et al. ^9^. **f)** *IRX4:NR2F2* ratios for human and rhesus iPSC-CMs and the three earliest matching prenatal hearts from Cardoso-Moreira et al. ^9^. Source data

Motivated by previous findings on specific hallmarks of transcriptional and translational evolution during organ development^26^, we reprogrammed human, chimpanzee, gorilla and rhesus fibroblasts to iPSC lines and later differentiated to iPSC-CMs to represent early fetal-stage cardiac cells^10,30^ (Fig. 2a, Extended Data Fig. 2a,b and Supplementary Table 1). We next performed Ribo-seq and matched RNA-seq to profile the generated iPSC-CMs (Fig. 2b and Extended Data Fig. 1a–d). We identified an average of 11,610 transcribed genes (replicated in at least three samples), of which 9,586 were translated (Methods). Principal component analysis (PCA) of both transcribed and translated genes progressively delineated adult LV, iPSC-CM and iPSC samples similarly across all species, suggesting a conserved developmental order (Fig. 2c,d). The differences in gene expression between iPSC-CMs and adult LVs and between prenatal and postnatal hearts from a previously published study^9^ were highly correlated (rho = 0.72–0.76; Fig. 2e–g and Extended Data Fig. 2c).

The Ribo-seq profiles of human, chimpanzee and gorilla iPSC-CMs revealed a low *TNNI3:TNNI1* ratio and a high *MYH7:MYH6* ratio, suggesting a predominantly fetal ventricular identity^29^ (Extended Data Fig. 2d). Rhesus iPSC-CMs differed slightly, having a unique inverted ratio of *MYH7:MYH6*, which, in humans, would generally suggest a more atrial identity^29^. Although some dissimilarities observed between rhesus and the other species could potentially stem from variations in the differentiation protocols, our analysis of publicly available prenatal rhesus samples^9^ revealed a similar inverted *MYH7:MYH6* ratio in rhesus compared to human hearts (Extended Data Fig. 2e), indicating that the observed differences are also reflecting species-specific distinctions. In addition, the *TNNI3:TNNI1* ratio remained consistent across rhesus and human iPSC-CMs and prenatal hearts. Comparison of the wider profiles revealed that all iPSC-CMs expressed a mixture of markers predominantly indicating early developmental-stage cardiac cell types. Rhesus iPSC-CMs again diverged with higher expression of atrial markers, such as *NR2F2* (Fig. 2f). Accordingly, prenatal rhesus hearts also showed a more atrial identity defined by higher levels of *NR2F2* compared to ventricle-specific *IRX4* (Extended Data Fig. 2f).

Taken together, these data indicate that primate iPSC-CMs can serve as a model of fetal hearts, with rhesus iPSC-CMs displaying a more atrial identity.

### Emergence of young genes across primate cardiac development

**Fig. 3:**
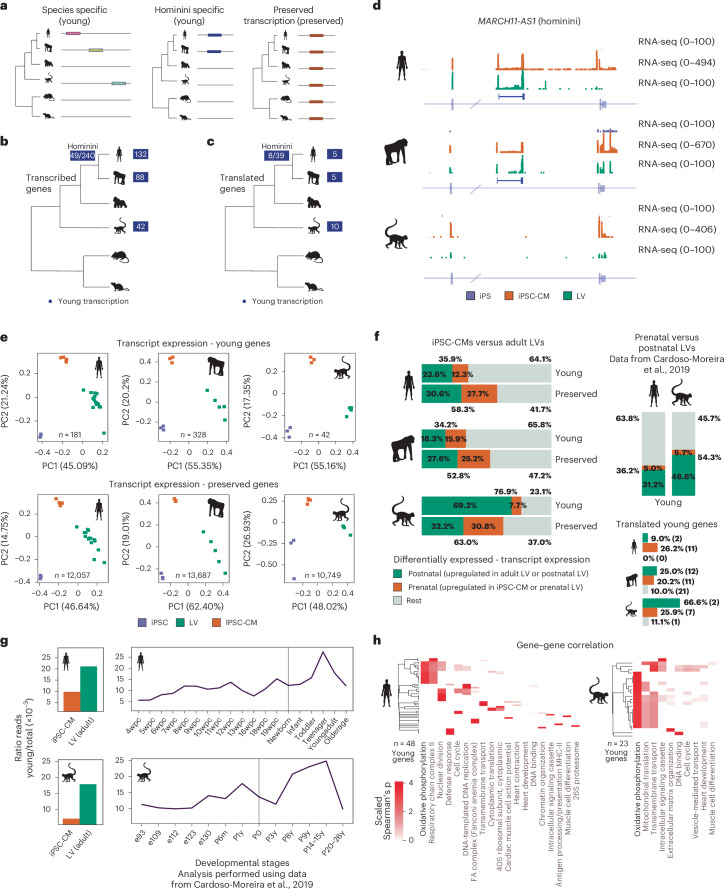
Emergence of new genes across cardiac development. **a**–**d**, Schematic and estimated number of transcribed (**b**) and translated (**c**) evolutionarily young genes in human, chimpanzee and rhesus. Hominini refers to the lineage comprising both humans and chimpanzees. An example of an intronic hominini-specific gene antisense to *MARCH11* is shown in **d**. Only species with both iPSC-CM and LV data are shown. The gene is not expressed in gorilla, mouse and rat. **e**, PCA of the transcript expression levels of evolutionarily young (top) and transcriptionally preserved (bottom) genes across iPSCs, iPSC-CMs and adult LVs. **f**, Percentages of preserved and young genes that are differentially transcribed between iPSC-CMs and adult LVs (left) or between prenatal and postnatal LVs (top right; data from Cardoso-Moreira et al.^9^) across species, representing genes enriched prenatally and postnatally, respectively. Bottom right, percentages and numbers of young translated genes by directionality of regulation in transcription. **g**, Distribution of the ratios of the number of reads mapping to human and rhesus young genes for pooled iPSC-CM and adult LV datasets (left) and by developmental stage (analysis performed using data from Cardoso-Moreira et al.^9^) (right). Vertical lines mark birth. **h**, Enriched CORUM and Gene Ontology terms of genes highly correlating to human and rhesus young genes in genome-wide gene–gene transcription correlations across cardiac development (analysis performed using data from Cardoso-Moreira et al.^9^). PC, principal component; wpc, weeks post conception. Source data

The emergence of new genes is fundamental to the evolution of tissue complexity^31–33^. We evaluated the contribution of newly evolved genes to the transcriptomes of iPSC-CMs and adult hearts. First, we assessed whether transcribed genes (protein coding, lncRNA, pseudogene and unannotated) identified in human, chimpanzee and rhesus iPSC-CMs and adult LVs were preserved across species by searching for transcribed homologous sequences (Fig. 3a and Methods). Among these species, 132 human, 88 chimpanzee and 42 rhesus genes showed species-specific transcription (Fig. 3b). We also found 49 human and 240 chimpanzee genes that emerged during hominini evolution around 6–8 Mya. In total, we identified 12 protein-coding, 45 lncRNA and 494 unannotated young genes without preserved transcription (Supplementary Tables 2–4). Only 20 species-specific and 47 hominini-specific genes showed translational activity in at least one of the three species, all without annotated functions (Fig. 3c). One example is a young antisense gene emerged within the intron of *MARCH11* (or *MARCHF11*) in hominini (Fig. 3d).

PCA revealed that the two first principal components explained most of the variance in transcribed young gene expression among iPSCs, iPSC-CMs and adult LVs (Fig. 3e). However, differentially expressed young genes in humans and chimpanzees were less prevalent than in older genes (35.9% and 34.2% for young genes, 58.3% and 52.8% for transcriptionally preserved genes, *P*_human_ = 3.59 × 10^−9^, *P*_chimp_ = 1.90 × 10^−10^; Fig. 3f), indicating that the expression of evolutionarily preserved genes is more dynamically regulated across cardiac development. In contrast, rhesus had a significantly higher proportion of differentially transcribed evolutionarily young genes than older genes (76.9% for young genes, 63.0% for transcriptionally preserved genes, *P*_rhesus_ = 0.048). Reanalysis of human and rhesus cardiac developmental data^9^ showed similar trends, although to a lesser extent (Fig. 3f; *P*_human_ = 0.0001, *P*_rhesus_ = 0.009).

In the three primate species, adult LVs exhibited a higher abundance of differentially transcribed young genes than iPSC-CMs (23.6% versus 12.3% in human, 18.3% versus 15.9% in chimpanzee, 69.2% versus 7.7% in rhesus for LVs and iPSC-CMs, respectively), constituting a more substantial fraction of the overall transcriptome (Fig. 3f,g). We next reanalyzed human and rhesus cardiac developmental data^9^ and observed an increasing trend in the global expression of transcribed young genes throughout developmental time, with a decline observed at the oldest developmental timepoint (Fig. 3g).

Next, we clustered genome-wide transcription correlations to infer possible functional co-regulation of young genes. Consistent with our previous findings in human lncRNAs^15^, a subset of human and rhesus young genes correlated with protein-coding genes involved in OXPHOS, among other functions (Fig. 3h). This observation is intriguing, considering that human and rhesus young genes emerged independently in distinct lineages and are located in different genomic regions. This suggests the possibility of shared, recently co-evolved regulatory mechanisms.

Collectively, our data support the presence of transcriptional events specifically regulated in iPSC-CMs and adult LVs as well as across development, including evolutionarily young genes that may underlie species-specific differences between primate hearts.

### Recent evolution of human cardiac-enriched genes

**Fig. 4:**
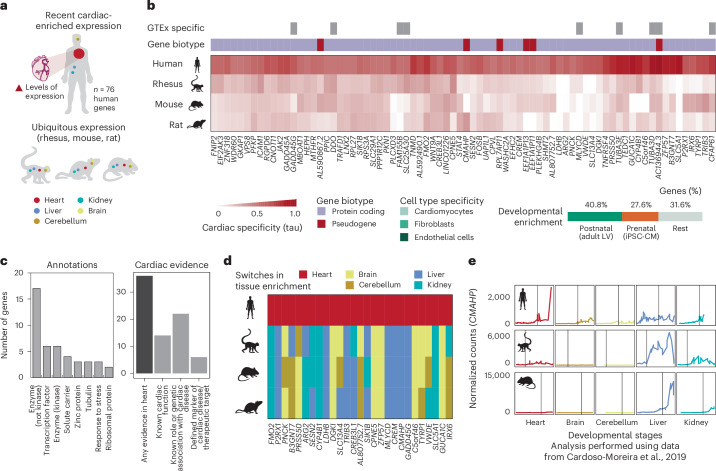
Recent evolution of human cardiac-enriched genes. **a**,**b**, Schematic and heatmap with the levels of tissue enrichment of genes with recently acquired enrichment of cardiac expression in humans (*n* = 76, analysis performed using data from Cardoso-Moreira et al.^9^). Gene biotypes and cardiac/muscle specificity in GTEx are represented with different colors. **c**, Annotated functional descriptions and compiled evidence of functions or associations with cardiac function and disease for the 76 human cardiac-enriched genes. Only descriptions annotated for two or more genes in Ensembl version 98 are displayed. **d**, Heatmap with human cardiac-enriched genes that are enriched in different tissues in other non-human species (*n* = 27). Colors indicate the tissue that the gene is specific to. **e**, Expression of human *CMAHP* and the orthologous rhesus and mouse *CMAH* across different organs and developmental stages (analysis performed using data from Cardoso-Moreira et al.^9^). Vertical lines mark birth. Human *CMAHP* recently acquired heart-enriched expression, whereas rhesus macaque and mouse *CMAH* is enriched in liver. Source data

The evolution of new developmental trajectories in conserved genes can also facilitate organ innovations^9^. By leveraging public developmental data for five organs (heart, brain, cerebellum, kidney and liver) from human, rhesus, mouse and rat^9^, we found 76 translated genes that, despite being evolutionarily conserved, recently acquired cardiac-enriched expression in humans (Fig. 4a,b, Supplementary Table 5 and Methods). Similar to what we observed for newly evolved genes (Fig. 3f), the expression of these genes was biased to the postnatal stage, with 40.8% being upregulated in adult LVs and 27.6% in iPSC-CMs (Fig. 4b). This set of genes comprises various functional categories, including transcription factors, solute carrier transporters, kinases and other proteins with enzymatic capacity (Fig. 4c). By curating the literature, we could assign 36 of 76 genes to published cardiac evidence (Fig. 4c and Methods). Of these, 14 genes are already described to exert a function in the heart; 22 have a known role or genetic association with cardiac disease; and six have been proposed as potential cardiac biomarkers or therapeutic targets (Supplementary Table 5).

**Extended Data Fig. 3:**
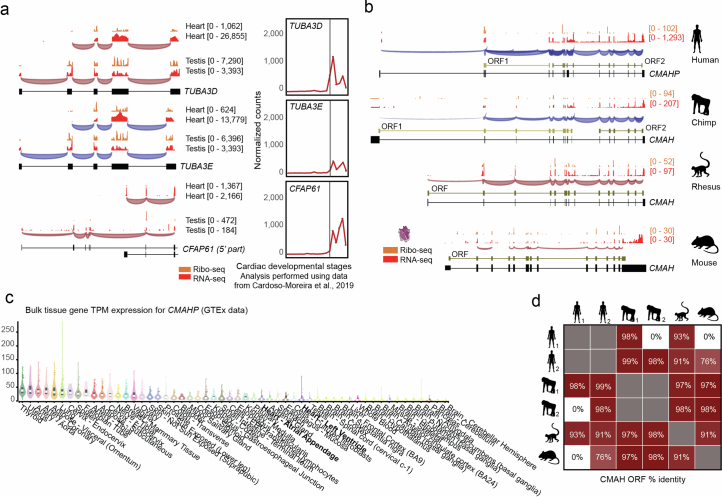
Recent evolution of human cardiac-enriched genes. **a)** Human adult LV (this study) and testis (Wang et al. ^26^) Ribo-seq and RNA-seq profiles (left) and normalized RNA-seq expression levels across cardiac developmental stages (Analysis performed using data from Cardoso-Moreira et al. ^9^, right) of three genes with human-specific heart/muscle enrichment and with unknown cardiac function. Vertical lines mark birth. For *CFAP61*, only the 5′ region of the gene is represented. **b)** Genomic tracks of Ribo-seq, RNA-seq, RNA-seq junctions, and predicted open reading frames (ORFs) for the human pseudogene *CMAHP* and its orthologous protein-coding genes *CMAH* in chimp, rhesus, and mouse. Pooled data of LV samples are shown. **c)** TPM expression of *CMAHP* across human tissues in GTEx^51^. Tissues are sorted by median TPM expression. **d)** Percentage of identity of predicted ORFs in *CMAHP/CMAH* across four species. Source data

We also inspected the tissue specificity of these genes in a broader catalog of 54 human tissues (Genotype-Tissue Expression (GTEx))^34^. We found that nine of 76 genes were specific to the heart and/or muscle in GTEx (Supplementary Table 5). Of these, AC136944.3 likely emerged due to a partial duplication of *CHK2*, whereas *TUBA3D*, *TUBA3E* and *CFAP61* were highly expressed in mammalian testis and acquired new promoters encoding for human alternative cardiac isoforms (Extended Data Fig. 3a). None of these genes has reported cardiac functions and is highly expressed postnatally.

Of note, 27 human cardiac-enriched genes exhibited enrichment in a non-cardiac tissue in the other species, suggesting a switch in the patterns of tissue expression across human evolution (Fig. 4d). One intriguing example with human-specific, cardiac-enriched expression is the *CMAH* pseudogene (*CMAHP*) (Fig. 4d,e and Extended Data Fig. 3b). In various non-human species, the *CMAH* gene encodes a cytidine monophosphate-*N*-acetylneuraminic acid hydroxylase, which is primarily expressed in the liver and involved in the conversion of sialic acids. In humans, a loss-of-function mutation occurred in this gene approximately 2–3 Mya, resulting in its pseudogenization^24^, but the gene is still expressed ubiquitously across human tissues (Extended Data Fig. 3c). We found that the human *CMAHP* locus encodes two distinct translated ORFs in the adult heart, one of which shares partial homology with the CMAH CDS found in NHPs and rodents (Extended Data Fig. 3d). Rather than being a non-functional degenerated pseudogene, the recent acquisition of expression in adult human hearts suggests a potential role either for the RNA or the two ORFs it encodes.

Thus, these results indicate that a subset of conserved human genes recently acquired new tissue expression patterns in the heart and may play species-specific roles in development.

### Translation of evolutionarily young sORFs in primate hearts

**Fig. 5:**
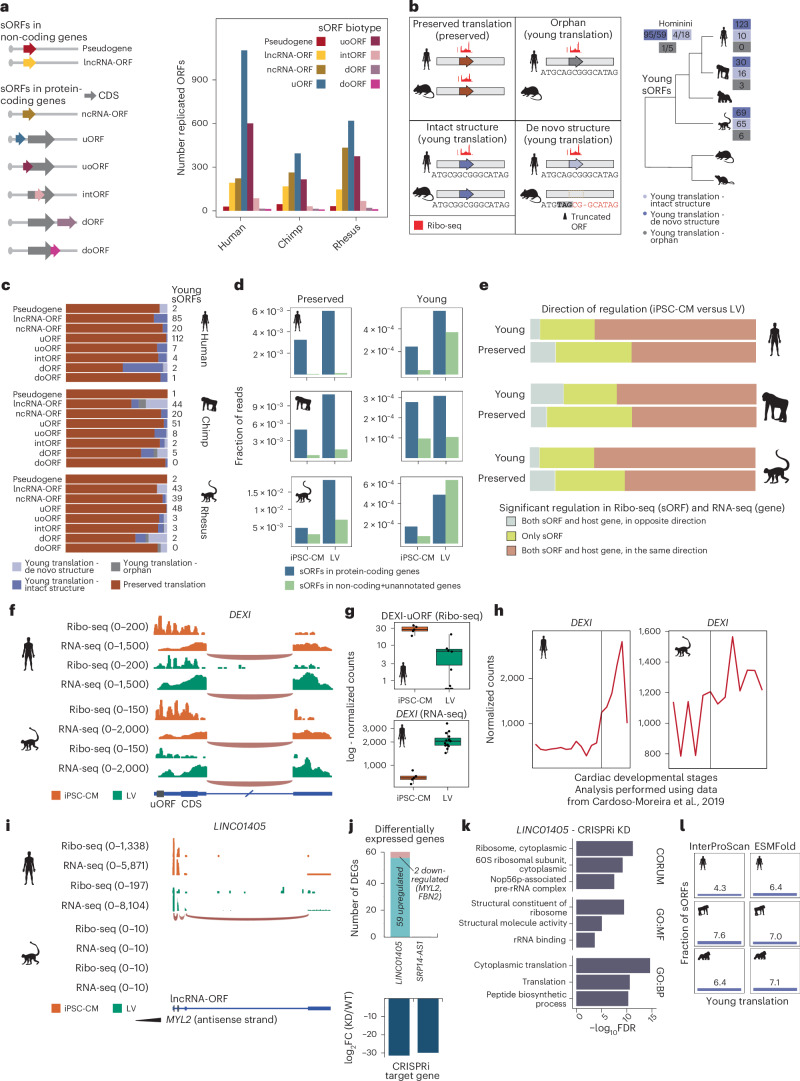
Translation and evolution of sORFs across primate cardiac development. **a**, Left, schematic of the sORF biotypes considered in this study. Right, number of replicated sORFs per species and biotype. **b**, Left, schematic of the evolutionary age of sORFs based on the presence or absence of translation across species and on the presence or absence of ORF structures for cases with young translation. Right, number of young sORFs with intact structure (blue), de novo structure (light blue) or without aligned counterpart sequences (orphan, in gray). **c**, Proportions of sORFs by biotype and age classification. Numbers of young sORFs are shown. **d**, Fraction of Ribo-seq reads mapped to sORFs in non-coding or protein-coding genes, separated by developmental stage, age and species. **e**, Percentages of young and preserved sORFs with similar or opposite direction of translational regulation between iPSC-CMs and adult LVs, compared to the transcription of their host genes. **f**, An example of a uORF upregulated in iPSC-CMs encoded by *DEXI*. Counts are normalized for visualization. **g**, Box plots with normalized expression levels of *DEXI* (RNA-seq) and its uORF (Ribo-seq) across human iPSC-CM (*n* = 5) and adult LV (*n* = 15) samples. Boxes indicate interquartile range (IQR; 25th and 75th percentiles); center line indicates median; and whiskers indicate minimum to maximum. **h**, Normalized RNA-seq expression levels of *DEXI* across human and rhesus cardiac developmental stages. Vertical lines mark birth. **i**, An example of a hominini-specific lncRNA-ORF encoded by the *LINC01405* (or *STRG-HSA-132505*) gene. Counts are normalized for visualization. Because *SRP14-AS1* did not result in any observable phenotype, we did not include this gene for visualization. **j**, Top, number of differentially expressed genes after perturbing *LINC01405* and *SRP14-AS1* expression with CRISPRi. Bottom, magnitude of change in expression of the target gene by CRISPRi for knockdown (KD). Both genes showed significant downregulation after KD: adjusted *P* = 3.7 × 10^−4^ and *P* = 2.6 × 10^−6^ for *LINC01405* and *SRP14-AS1*, respectively. **k**, CORUM and Gene Ontology terms significantly enriched in the group of genes affected after genetic perturbation of *LINC01405* in iPSC-CMs. CRISPRi, clustered regularly interspaced short palindromic repeats (CRISPR) interference. **l**, Fraction of young sORFs with predicted InterProScan motifs and ESMFold structures. DEG, differentially expressed gene; FC, fold change; GO:BP, Gene Ontology: Biological Proccess; GO:MF, Gene Ontology: Molecular Function. lncRNA-ORF, ORF in long non-coding RNA or unnanotated gene; ncRNA-ORF, ORF in non-coding RNA isoform; uoORF, upstream overlapping ORF; intORF, internal ORF; dORF, downstream ORF; doORF, downstream overlapping ORF. Source data

**Extended Data Fig. 4:**
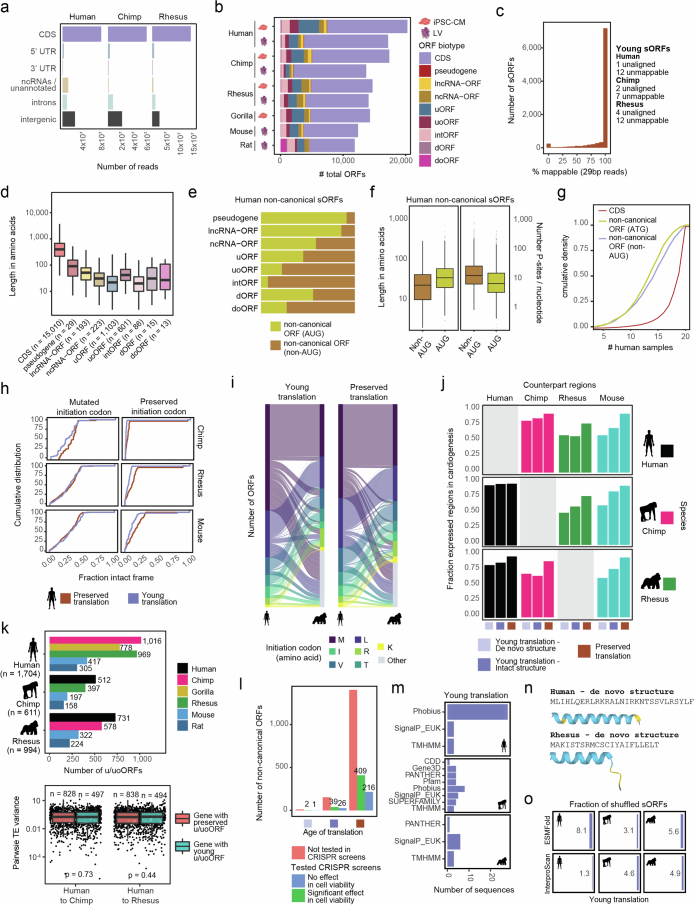
Translation and evolution of sORFs during cardiac development. **a)** Number of Ribo-seq reads mapped to features of protein-coding and non-coding genes (annotated and unannotated). **b)** Number of predicted ORFs per sample and dataset, divided by ORF biotype and including CDSs. **c)** Histogram depicting the number of human, chimp, and rhesus sORF sequences with young translation mappable by reads of 29 bp (average size of ribosome-protected fragments) across the other primate genomes considered in the study. The accompanying text describes the count of young sORFs that remain unmappable or unaligned to any other primate genome. **d)** Distribution of human sORF lengths for each biotype. **e)** Fractions of cognate (n = 905) and non-AUG (n = 1,358) human sORFs per ORF biotype. **f)** Distribution of human ORF lengths and P-sites per nucleotide for cognate and non-AUG sORFs. **g)** Cumulative density of the replicability of CDSs, cognate AUG sORFs, and non-AUG sORFs across human samples. **h)** Cumulative density of the fraction of intact frame in human recent and preserved sORFs with mutated or preserved initiation codon in the other species. **i)** Proportion of initiation codons in human ORFs with recent and preserved translation and their counterpart regions in rhesus. **j)** Fraction of counterpart ORF regions transcribed in other species based on RNA-seq data, divided by levels of ORF age. **k)** Top: Number of primate uORFs/uoORFs with or without preserved translation in other species. Bottom: Distribution of pairwise TE variance of human CDSs hosting uORFs/uoORFs preserved or not preserved in chimp or in rhesus. Two-sided Wilcoxon rank-sum test adjusted p-values are displayed. **l)** Number of sORFs tested in published CRISPR screens, including cases that showed a significant effect on cell viability. ORFs are separated by the age of translation. **m)** Number of predicted InterproScan motifs in sORFs by signature and species. **n)** Two examples of *de novo* sORFs with predicted alpha helices by ESMFold in human and rhesus. **o)** Fraction of shuffled amino acid sequences with predicted InterproScan motifs and ESMFold structures. In panels **d)**, **f)**, and **k)**, boxes indicate interquartile range (IQR; 25th and 75th percentiles); center line indicates median ; whiskers indicate minimum to maximum. Source data

Translated sORFs outside CDS regions have emerged as important players in development and organogenesis^23,35,36^. Given the abundance of Ribo-seq reads in presumed non-coding regions, we integrated all LV and iPSC-CM Ribo-seq samples to predict translated cognate and non-AUG ORFs (Extended Data Fig. 4a–g and Methods). We focused on three subsets of replicated sORFs in humans, chimpanzees and rhesus, comprising 2,263, 1,143 and 1,706 sORFs, respectively (Fig. 5a and Methods). These sORFs, averaging 43 amino acids in length, were encoded by pseudogenes, non-coding RNAs and UTRs and did not overlap with in-frame CDSs (Fig. 5a and Extended Data Fig. 4d).

We next evaluated the evolution of these translated sORFs (Fig. 5b and Supplementary Table 6), identifying 322 and 182 young sORFs with preserved species-specific and hominini-specific translation, respectively. Most young sORFs were found in 5′ UTRs (upstream ORFs (uORFs)) or non-coding transcripts (lncRNA-ORFs and ncRNA-ORFs) (Fig. 5c). Young sORFs exhibited lower preservation of translation initiation sites but similar frame preservation across untranslated counterpart sequences (Extended Data Fig. 4h,i). This observation is likely not solely because of protein-coding constraints but due to high sequence similarity between primate genomes.

A minimum fraction of young sORFs was encoded by genomic regions without aligned counterparts in the other species (‘orphan’, 2.98%, *n* = 15; Fig. 5b), whereas most retained full structures in counterpart regions of other species (‘intact structures’, 74.01%, *n* = 376). More than half of these untranslated counterpart sequences were transcribed in other primate and rodent cardiac RNA-seq samples (Extended Data Fig. 4j). Hence, some sORFs might be still translated in other species in different tissues or conditions while remaining undetected in our datasets. Therefore, we used a modification of our previously published multi-species approach (Methods)^21^ to identify a subset of 113 young sORFs that presented truncated, untranslated ORF structures in the rest of analyzed primate and mammalian genomes, therefore likely emerging de novo (‘de novo structures’; Fig. 5b). Of these, 47 de novo sORFs were encoded by preserved non-coding genes and five by evolutionarily young genes.

Young sORFs with both preserved and young translation were more abundant in adult LVs compared to iPSC-CMs, similar to what we observed for the transcription of young genes (Fig. 5d). Although the changes in sORF translation were generally followed by changes in host gene mRNA levels, 25–40% of the sORFs displayed translation-specific regulation between iPSC-CMs and adult LVs, including nine young and 155 preserved ORFs with opposite regulatory trajectories compared to their host genes (Fig. 5e). An example is the uORF encoded by the dexamethasone-induced gene *DEXI*, which was highly translated in iPSC-CMs but downregulated in adult LVs, whereas its host transcript levels increased (Fig. 5f–h). uORFs can regulate the levels of translation of the downstream CDS, but our analysis did not reveal any statistically significant, genome-wide alterations in TE attributed to the emergence of young uORFs (Extended Data Fig. 4k).

Our findings indicate that sORFs with evolutionarily young cardiac translation are frequently translated, with a small fraction emerging de novo from ancestral non-coding regions.

### Functional roles of young sORFs translated in human hearts

We next sought to evaluate different lines of functional evidence of young sORFs. We found 41 young sORFs with previously demonstrated essential phenotype after knockout in human cell lines^37–40^ (Extended Data Fig. 4l), including *DEXI* uORF and two de novo sORFs encoded by the lncRNAs *LINC01128* and *ENSG00000288659* (or *STRG-HSA-40827*). Notably, we previously found a possible role for the microprotein encoded by *LINC01128* in intracellular trafficking and endocytosis^21^.

As an initial exploration of the potential impact of recently evolved sORFs in a cardiac context, we perturbed the expression of two human cardiac genes (*SRP14-AS1* and *LINC01405*) that emerged in primate evolution, that are absent in rhesus and that encode for young sORFs in iPSC-CMs (Fig. 5i and Methods). The disruption of *SRP14-AS1* in iPSC-CMs did not result in any significant changes in the transcriptome. In contrast, after the genetic repression of *LINC01405*, a cardiac-enriched lncRNA antisense to *MYL2*, we observed upregulation of 59 genes in iPSC-CMs, with a notable enrichment in translation and ribosome biogenesis (Fig. 5j,k). Only its sense gene *MYL2* (log_2_ fold change = −4.65) and *FBN2* (log_2_ fold change = −1.94) showed downregulation after disruption. Of note, a previous study implicated this gene in the regulation of cardiomyocyte specification, and its knockdown also led to the downregulation of *MYL2* (ref. ^41^).

We next screened for domains and structures in the putative microproteins encoded by human young sORFs (Fig. 5l and Methods). As expected, given the recent evolutionary origin of these sORFs, only a small fraction encoded microproteins with known domains (4.3–7.6% per species, totaling 29 sORFs, including six de novo structures) or with accurately predicted structures (6.4–7.1% per species, totaling 34 sORFs, including three de novo structures). These predictions mainly included signal peptides and alpha helices (Extended Data Fig. 4m,n). Notably, these proportions were not significantly different from shuffled sequences with similar amino acid composition (1.3–4.9% for known domains, 5.6–8.1% for predicted structures, *P* = 0.06–0.44; Extended Data Fig. 4o).

Together, these data suggest that at least a handful of evolutionarily young sORFs may encode structured, functional microproteins.

### Human cardiac genomic innovations are dysregulated in disease

**Fig. 6:**
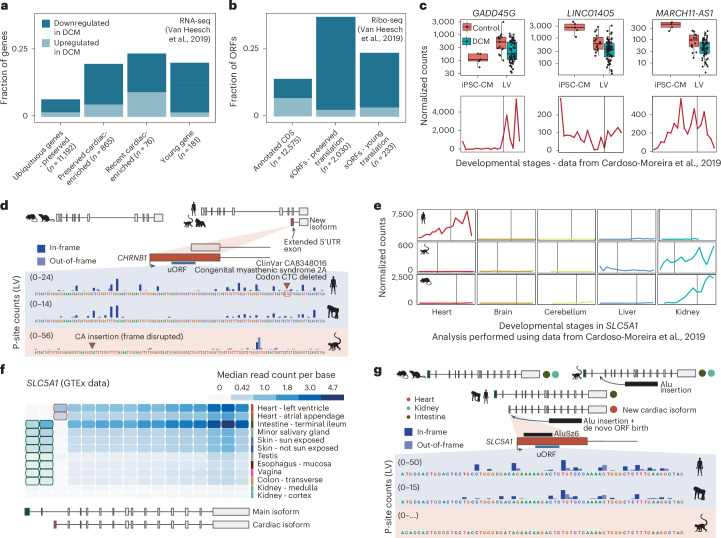
Dysregulation of recently evolved or recently enriched cardiac genes and sORFs in disease. **a**, Fraction of ubiquitous preserved genes, preserved cardiac-enriched genes, recent cardiac-enriched genes and young genes that are differentially expressed in DCM in humans. **b**, Fraction of human sORFs with preserved or young translation and CDS that are differentially translated in DCM. **c**, Normalized RNA-seq expression levels of three example genes—*GADD45G* (recent cardiac-enriched expression in humans), *LINC1405* (primate gene encoding a young sORF) and *MARCH11-AS1* (young hominini gene encoding a young sORF; Fig. 3d)—in iPSC-CMs (*n* = 5), adult LVs (*n* = 15) and diseased DCM LVs (*n* = 65) (analysis performed using RNA-seq data from Van Heesch et al.^15^) as well as across cardiac developmental stages (analysis performed using data from Cardoso-Moreira et al.^9^). All samples per species represent biological replicates. Vertical lines represent birth. Boxes indicate interquartile range (IQR; 25th and 75th percentiles); center line indicates median; and whiskers indicate minimum to maximum. All genes are significantly dysregulated in DCM (*GADD45G* DESeq2 adjusted *P* = 0.0007; *LINC01405* DESeq2 adjusted *P* = 0.0363; *MARCH11-AS1* DESeq2 adjusted *P* = 0.0020). **d**, *CHRNB1* contains a young translated uORF in a new primate-specific isoform with an extended 5′ UTR. P-site coverage of adult LVs for human, chimpanzee and rhesus is displayed. This uORF overlaps a ClinVar variant that deletes a CTC codon and is associated with congenital myasthenic syndrome 2A. **e**, RNA-seq expression of human *SLC5A1* and orthologous rhesus and mouse genes across organs and developmental stages. Vertical lines mark birth. Human *SLC5A1* recently acquired cardiac expression, whereas rhesus macaque and mouse *SLC5A1* is expressed in the kidney. **f**, Exonic expression of *SLC5A1* across human tissues in GTEx data. Tissues without detected expression are not displayed, except for the kidney. **g**, Exon 3 of *SLC5A1* is cardiac specific and emerged in Old World monkeys; it is partially derived from an AluSz6 element and contains a translated de novo uORF in hominini. P-site coverage of adult LVs for human, chimpanzee and rhesus is displayed. Source data

**Extended Data Fig. 5:**
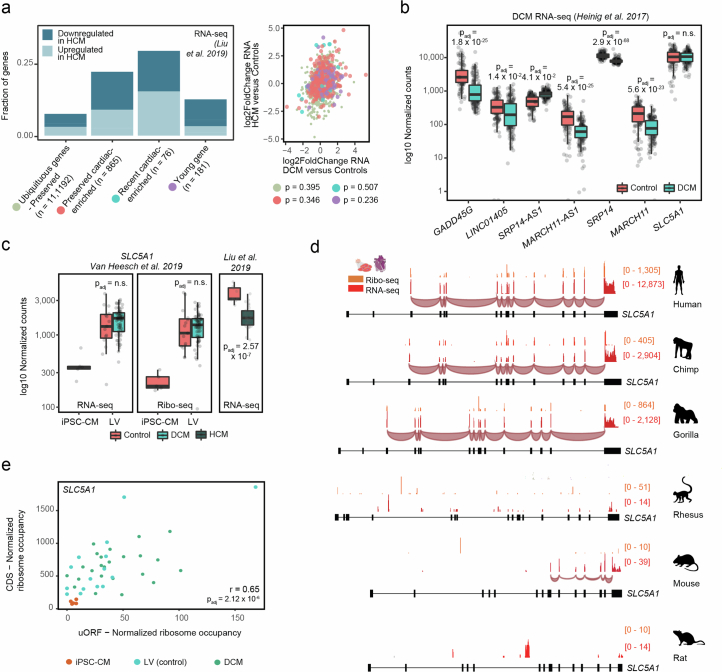
Dysregulation of recently evolved or recently enriched cardiac genes and sORFs in disease. **a)** Left: Fraction of ubiquitous preserved genes, preserved cardiac-enriched genes, recent cardiac-enriched genes, and evolutionarily young genes that are differentially expressed in hypertrophic cardiomyopathy (HCM, 9 controls and 28 disease donors) in humans. Right: Correlation between the log2 FoldChanges of mRNA abundance between dilated cardiomyopathy (DCM) and controls, and between HCM and controls, for both analyzed cohorts. Spearman’s rank correlation coefficients by gene group are also displayed. **b)** Box plots with normalized RNA-seq expression values of seven selected genes in a cohort of 97 control and 108 dilated cardiomyopathy (DCM) donors (Biological replicates, analysis performed using data from Heinig et al. ^44^). Boxes indicate interquartile range (IQR; 25th and 75th percentiles); center line indicates median (50th percentile); whiskers indicate minimum to maximum. *MARCH11-AS1* is regulated in the same direction as its sense gene *MARCH11*, whereas *SRP14-AS1* and its sense gene *SRP14* show opposite regulation in DCM, pointing to different locus-specific regulatory mechanisms. Adjusted p-values are calculated by DESeq2. **c)** Box plots with normalized expression values of *SLC5A1* in HCM and in a cohort of 15 control and 65 dilated cardiomyopathy (DCM) donors (Biological replicates, analysis performed using data from Van Heesch et al. ^15^). Boxes indicate interquartile range (IQR; 25th and 75th percentiles); center line indicates median (50th percentile); whiskers indicate minimum to maximum. Adjusted p-values are calculated by DESeq2. **d)** Genomic tracks of Ribo-seq, RNA-seq, and RNA-seq junctions for *SLC5A1* in human, chimp, gorilla, rhesus, mouse and rat. Pooled data of iPSC-CM and LV samples are shown. **e)** Pearson’s correlation of the Deseq2-normalized levels of ribosome occupancy between *SLC5A1* uORF and CDS for iPSC-CM, adult LVs, and DCM samples. The uORF and the CDS show a significant positive one-sided correlation. Source data

We next asked whether the abundance and translation levels of the three classes of new human cardiac genomic innovations, namely young sORFs, young genes and genes with human-specific cardiac-enriched expression, were affected in disease. Reanalyzing RNA-seq and Ribo-seq data from the LVs of 65 patients with end-stage dilated cardiomyopathy (DCM)^15^, we found that human young genes and genes with recently acquired cardiac-enriched expression had similar dysregulation frequencies as other cardiac-enriched genes (19.3% for young genes, 23.7% for genes with human cardiac-enriched expression, 16.5% for the rest of cardiac-enriched genes, *P* ≥ 0.24; Fig. 6a). In contrast, ubiquitous genes were less frequently dysregulated (6.1%, *P* ≤ 1 × 10⁻^4^). This observation is not specific to DCM, because similar fractions of genes were dysregulated in hypertrophic cardiomyopathy (HCM)^42^ (Extended Data Fig. 5a). Similar trends were observed for the translation of sORFs, with 39.0% of preserved and 24.9% of young sORFs dysregulated in DCM compared to 13.4% of annotated CDSs (*P* ≤ 2 × 10^−15^; Fig. 6b).

Notably, the vast majority of evolutionarily young human genes dysregulated in DCM were found to be aberrantly downregulated (91.7%; Fig. 6a). Similarly, most young sORFs were also downregulated (86.0%; Fig. 6b). Among genes with recent cardiac-enriched expression that were dysregulated in DCM, 66.6% displayed decreased expression in the disease context. In contrast, for ubiquitous preserved genes and annotated CDSs affected by DCM, an average of 73.5% (ubiquitous preserved genes, RNA-seq) and 49.5% (annotated CDSs, Ribo-seq) were downregulated in disease (Fig. 6a,b).

Genes dysregulated in DCM included the human growth arrest and DNA-damage-inducible 45 gamma (*GADD45G*), a regulator of cardiomyocyte death^43^ that acquired cardiac-enriched expression in adult LVs (Fig. 6c). Three examples of primate-specific genes dysregulated in DCM that encode sORFs with young translation are the above-described *MARCH11-AS1*, *SRP14-AS1* and *LINC01405* (Figs. 3d, 5i and 6c). Consistently, the dysregulation of these genes was replicated in another DCM cohort^44^ (Extended Data Fig. 5b).

We further found two young sORFs, 23 young genes and 19 genes with human-specific cardiac-enriched expression harboring pathogenic or likely pathogenic ClinVar^45^ variants (Supplementary Table 7). Only eight of the young genes were found to have variants directly associated with cardiac disease (Methods), although other mapped variants are implicated in developmental disorders and may also impact cardiac function. One example is a young uORF encoded by *CHRNB1* (cholinergic receptor nicotinic beta 1 subunit). This gene is linked to slow-channel congenital myasthenic syndrome. Some subtypes of this disease are associated with higher heart rate variability and arrhythmia^46^. We discovered one pathogenic ClinVar mutation, initially annotated as intronic and potentially disrupting a consensus splice site, that deletes a CTC codon in the uORF of an alternative cardiac primate isoform (Fig. 6d).

Additionally, other genes and sORFs may be affected in cardiac conditions not covered in our study. For instance, *SLC5A1* (or *SGLT1*) is a sodium-glucose co-transporter with conserved expression in the intestine^47^. *SLC5A1* is downregulated in HCM but not in DCM (Extended Data Fig. 5c). Additionally, this gene is known to be upregulated in diabetic and ischemic cardiomyopathy and plays a role in myocardial energy metabolism^48,49^. In non-hominini mammals, the gene is highly expressed in the kidney (Fig. 6e). In humans and chimpanzees, *SLC5A1* lost its kidney expression and evolved cardiac expression due to the emergence of a cardiac-specific isoform with an alternative 5′ UTR exon, partially derived from an AluSz6 SINE element (Figs. 4b and 6f,g and Extended Data Fig. 5d). This transposable element was inserted into the genome of the Old World ancestor, followed by the de novo emergence of a hominini-specific upstream ORF that positively correlates with the translation levels of the main CDS (Fig. 6g and Extended Data Fig. 5d,e).

In conclusion, our findings reveal widespread dysregulated transcription and translation of numerous sORFs and genes exhibiting recently evolved cardiac expression patterns in cardiac disease.

## Discussion

In this study, we generated a comprehensive resource of transcription and translation of human and NHP LVs and established iPSC-CMs, uncovering regulatory dynamics of gene expression in primate evolution. Although recent efforts have been made to capture the entire genomic sequence diversity of NHPs^50^, the limited annotation of NHP genomes poses challenges for investigating differences in their transcriptomes and translatomes. To overcome this limitation, annotation-independent strategies have been followed to comprehensively characterize and compare the NHP transcriptomes^5,25^. We built a catalog of sORF translation that covers four primate species, considerably expanding the known primate cardiac translatomes.

Previous studies demonstrated translational control of gene expression in human adult hearts^15^ as well as during cardiac fibrosis^13^. The main genetic networks governing heart function are conserved across evolution^51^. However, in the context of recent mammalian evolution, we show that the TEs of nuclear-encoded OXPHOS complexes IV and V genes exhibit the fastest evolutionary rates. This rapid evolution may represent compensatory adaptations in response to changes in mRNA abundance, leading to relaxed selective constraints on transcript levels; a similar widespread phenomenon was previously observed in primate lymphoblastoid cell lines^52^. In addition, the fast evolution of translational control of nuclear-encoded OXPHOS genes might be a compensatory response to changes in mtDNA-encoded OXPHOS genes, ensuring stoichiometrically balanced synthesis^53^. Thus, the evolution of translational control across mammals might maintain mitochondrial oxidative metabolism, which is of pivotal importance for the adult heart^54^.

In our study, we further identified 1,626 primate cardiac genes displaying species-specific changes in TE across recent primate evolution. Many of these genes might potentially serve as previously unknown contributors to variations in heart physiology among primates and developmental stages. For instance, our analysis highlights a pronounced rhesus-specific increase in TE for components of mitochondrial translation, a process that controls cytoplasmic protein homeostasis^55^. Small primates, characterized by their high heart rates, necessitate increased energy production within cardiomyocytes^56^. Therefore, the observed adaptations in translational control may represent unknown contributors that facilitate species-specific adjustments in energy requirements within primates.

Furthermore, the additional inclusion of human and NHP immature iPSC-CMs as a model of early fetal cardiogenesis allowed us to identify species-specific differences in the transcription and translation of genes across cardiac development. For instance, we found that rhesus iPSC-CMs exhibit a more atrial identity compared to human, chimpanzee and gorilla iPSC-CMs. These rhesus-specific differences are characterized by lower levels of ventricle-specific *IRX4* relative to *NR2F2*, a critical gene for atrial differentiation, as well as a negative *MYH7:MYH6* ratio—*MYH7* being the major ventricular myosin heavy chain isoform in adult primate hearts. Notably, these observations are also mirrored in primate prenatal hearts.

Despite the high degree of similarity of primate genomes, we found that the heart organ is shaped by numerous recent transcriptional and translational events. We identified 551 and 504 young cardiac genes and sORFs—including 113 young sORFs that putatively emerged de novo from non-coding regions*—*as well as 76 conserved genes with human-specific, cardiac-enriched expression. Interestingly, we observed an increase in the expression of young genes and sORFs in postnatal hearts. Our findings reinforce a prior observation demonstrating that the expression of evolutionarily young genes increases during human development^9^ and spermatogenesis^8^, probably due to the stronger functional constraints acting in early development that hinder the expression of newly evolved genomic elements. Intriguingly, this seems to contradict previous reports indicating that the expression of young genes is biased toward prenatal development in the human brain^57^. We acknowledge that our comparative analysis is limited to the emergence of new genes and sORFs in the heart, but we cannot exclude that some genes and sORFs might be still transcribed and/or translated in other organs across longer evolutionary distances. A second limitation stems from the generation of iPSC-CMs from a single individual per species as a model of fetal cardiac development. Future analysis covering broader stages of prenatal and postnatal development across organs and including both male and female individuals will help to decipher how novel sORFs and genes orchestrate development in primates.

Although some of the genes and sORFs with recent origin or recently acquired expression in human hearts may be the result of pervasive, transient transcription and translation^20,25,58,59^, others may represent functional evolutionary innovations specific to primate species and may eventually be annotated as protein-coding genes. For example, young sORFs can encode microproteins that interact with proteins from conserved biological pathways^21^. Moreover, the disruption of young sORFs can affect cell fitness in cell line models^22,37–39^. In the present study, we observed many young genes integrated into OXPHOS co-regulation networks independently in humans and rhesus, suggesting the potential existence of a regulatory mechanism linked to energy metabolism. Moreover, a substantial fraction of human young genes, young sORFs as well as conserved genes with recently evolved cardiac-enriched expression were dysregulated in cardiomyopathies. We acknowledge that the observed widespread dysregulation does not necessarily mean that a causal relationship exists. However, prioritizing functional validation of these dysregulated genes and sORFs could reveal whether they have disease-promoting properties and whether their inhibition could represent a potential approach to treating heart failure. One promising case is *SLC5A1* (or *SGLT1*), a sodium-dependent glucose transporter whose levels of expression are increased in hypertrophic, diabetic and ischemic cardiomyopathy^48,49^. The dual inhibition of *SGLT1* and *SGLT2* can reduce the risk of heart failure hospitalizations^60^, although the mechanism is unclear. We detailed the evolution of cardiac *SGLT1* in primates, discovering an alternative isoform derived from an inserted transposable element that encodes a recently evolved uORF. This alternative isoform partially lacks a transporter domain in the N-terminus and does not participate in cardiac glucose handling^47^. Therefore, *SLC5A1* might exert its function in the heart using an unknown mechanism and specifically targeting cardiac *SLC5A1* isoform, and its encoded uORF might improve its protective cardiovascular effect and reduce potential side effects.

In summary, we described the evolutionary innovations in translational control, cardiac specificity and new gene and sORF emergence in human and NHP cardiac development. Our work constitutes the basis for future functional assays for studying the role of genes and sORFs in human cardiac physiology and disease.

## Methods

### Ethics statement

The tissue bank of the Biomedical Primate Research Centre (BPRC) complies with Article 4 (Principle of replacement, reduction, and refinement) and Article 47 (Alternative approaches) of Directive 2010/63/EU on the Protection of Animals Used for Scientific Purposes. The BPRC has been accredited by the Association for Assessment and Accreditation of Laboratory Animal Care since 2012. The generation of the iPSC line was accredited by the Ethikkommission der Universität Ulm (approval number: Antrag-Nr. 19/12).

### Collection of NHP tissues

Snap-frozen samples of heart LVs of four rhesus macaques (*Macaca mulatta*, three male and one female individuals) and five chimpanzees (*Pan troglodytes*, three male and two female individuals) were provided from the NHP tissue bank at the BPRC in Rijswijk, The Netherlands (Supplementary Table 1). The tissues from rhesus macaques were obtained from BPRC animals that were euthanized for welfare and ethical reasons. The chimpanzees’ tissues were derived from animals residing at Safari Park Beekse Bergen and on which necropsies were done at the BPRC in The Netherlands.

### Tissue processing

Snap-frozen NHP cardiac tissues were powdered using a pre-cooled mortar and pestle while constantly introducing liquid nitrogen^15^. This procedure was performed on days when humidity levels were below 30%. For each sample, we ideally reserved approximately 100 mg of powdered tissue (with a minimum of 50 mg) for ribosome profiling. Additionally, a smaller amount of 5–10 mg was gathered for the purpose of total RNA extraction and subsequent RNA-seq analysis.

### Human and NHP iPSC reprogramming

Dermal fibroblasts from human and NHP individuals with no known cardiovascular disorder or phenotype were reprogrammed to iPSCs using CytoTune 2.0 iPS-sendai virus (Thermo Fisher Scientific). A female human subject was recruited as part of a study at the Charité, Berlin, with broad consent given for human iPSC line generation and use for research purposes. A skin punch biopsy was taken, from which dermal fibroblasts were outgrown and cultured for several passages. Similarly, dermal fibroblasts were outgrown from skin punch biopsies taken from a female macaque (courtesy of Göttingen DPZ), a male gorilla (Rostock Zoo) and a male chimpanzee (Magdeburg Zoo). After colony outgrowth and isolation, clonal iPSC lines with stable uniform morphology were established under feeder-free conditions on Geltrex (Thermo Fisher Scientific) in E8 (Thermo Fisher Scientific) or mTeSR1 (STEMCELL Technologies) pluripotency media (Supplementary Table 1). This step could be performed directly with human (*Homo sapiens*), chimpanzee (*P. troglodytes*) and gorilla (*Gorilla gorilla*) iPSCs; however, rhesus (*M. mulatta*) iPSCs required an intermediate culture step on mitomycin C-inactivated mouse embryonic fibroblasts in an adapted pluripotency maintenance medium, based on StemMACS iPS-Brew (Miltenyi Biotec) supplemented with 1 µM IWR-1 (Selleck Chemicals) and 0.5 µM CHIR99021 (CHIR, Bio-Techne), referred to as ‘StemMACS UPPS’ from Stauske^61^. Several passages after this, the rhesus iPSC colonies were non-enzymatically dissociated and transferred to feeder-free conditions on Geltrex-coated plates in StemMACS UPPS for further culture, displaying a stable epithelial cell morphology and growth rate.

Consistent expression of pluripotency factors was confirmed in all human and NHP iPSC lines (see ‘Immunocytochemistry’ subsection; Extended Data Fig. 2a).

### IPSC-CM differentiation

Between three and five targeted iPSC-CM differentiation experiments were carried out for each species’ iPSC line. For human, gorilla and chimpanzee iPSCs, an adapted version of established protocols modulating WNT signaling using small-molecule inhibitors could be employed^62,63^, with minor adjustments in GSK3β-inhibitor CHIR99021 (CHIR, Bio-Techne) concentration and starting cell density needed between lines to achieve efficient differentiation (Fig. 2a and Supplementary Table 1).

In brief, iPSCs were enzymatically dissociated using TrypLE (Thermo Fisher Scientific) or non-enzymatically (gorilla iPSCs) and replated at 2–5 × 10^5^ cells per cm^2^ (or 1:6 split ratio for gorilla) on Geltrex-coated plates in pluripotency medium containing 10 μM Rho-kinase inhibitor Y-27632 (Selleck Chemicals). After 3–5 d, differentiation to mesoderm was initiated by exchanging for RPMI-based medium containing B-27 minus insulin (Thermo Fisher Scientific) and CHIR (differentiation ‘day 0’). After 3 d, CHIR was replaced with 5 μM WNT-inhibitor IWR-1 (Selleck Chemicals) to drive cardiac specification. After 7 d, CM maintenance medium comprising RPMI plus B-27 was used for all CMs, except during selection; after approximately 10 d of differentiation, iPSC-CMs underwent enrichment via metabolic selection, replacing glucose with sodium lactate (Sigma-Aldrich) for 2–3 d^64^.

For rhesus iPSCs, we found the initial stages of the differentiation protocol insufficient for generating iPSC-CMs with suitably high efficiency (data not shown), so we employed a strategy from Stauske^61^. This adapted approach included 5 ng ml^−1^ BMP4 and 9 ng ml^−1^ Activin A (both PeproTech) together with CHIR during mesodermal differentiation (Supplementary Table 1).

After approximately 2 weeks of differentiation, iPSC-CMs were dissociated using 10× TrypLE (Thermo Fisher Scientific) and replated at 2.5–3 × 10^5^ cells per cm^2^ onto fresh Geltrex-coated plates. After approximately 4 weeks of differentiation, iPSC-CMs were collected for quality control (see ‘Immunocytochemistry’ and ‘Flow cytometry’ subsections) or snap frozen as cell pellets.

After 4 weeks, human and NHP iPSC-CMs expressed classic cardiac markers, such as the transcription factor *NKX2-5* and *TNNT2* (cardiac troponin T), and variable expression of ventricular-specific *MYL2* (myosin light chain 2), higher in gorilla iPSC-CMs and lower in rhesus (Fig. 2a and Extended Data Fig. 2b).

### Immunocytochemistry

Cells were plated onto chamber slides (ibidi) pre-coated with Geltrex. After several days, cells were fixed with 4% paraformaldehyde (Science Services), washed with PBS and incubated with blocking solution containing 10% normal donkey or goat serum (Abcam) and 0.1% Triton X-100 (Sigma-Aldrich) in PBS with 0.05% Tween 20 (Sigma-Aldrich). Primary and secondary antibodies were used for labeling (Supplementary Table 8), followed by DAPI for nuclear counterstain. Microscopy was performed using a DMi8 microscope fitted with a K5 camera and processed using LASX software (all from Leica).

### Flow cytometry

Cells were harvested using 10× TrypLE (Thermo Fisher Scientific), stained for viability using VioBility Blue (Miltenyi Biotec), fixed and permeabilized using FoxP3 staining buffer kit (Miltenyi Biotec) and stained with conjugated antibodies (Supplementary Table 8). Population gates were set for whole single live cells, with positive gates for expression targets set based on isotype antibody staining. Expression was analyzed using a MACSQuant VYB flow cytometer (Miltenyi Biotec) with gating and plots visualized using FlowJo 10 software. A figure exemplifying the gate strategy is included in Supplementary Data 3.

### Ribosome profiling

In this study, we performed ribosome profiling on primate iPSC-CM cells and LVs, following established protocols described by our group and others^15,65–67^. In brief, a total of 9–15 million cells were lysed on ice for 10 min in 1 ml of lysis buffer. The lysis buffer composition was as follows: 20 mM Tris-Cl (pH 7.4), 150 mM NaCl, 5 mM MgCl_2_, 1% Triton X-100, 0.1% NP-40, 1 mM dithiothreitol, 10 U ml^−1^ DNase I, 0.1 mg ml^−1^ cycloheximide and nuclease-free water. To ensure complete lysis of the cells and to dissociate cell clumps, the lysate was homogenized through repeated pipetting and multiple passes using a syringe equipped with a 21-gauge needle. After homogenization, the samples were centrifuged at 20,000*g* for 10 min at 4 °C to pellet cellular debris. For each sample, 200 µl (cells) or 400 μl (tissue) of the lysate was digested with RNase 1 (Biozym, N6904K) and purified using MicroSpin Sephacryl S-400 HR columns (Sigma-Aldrich, GE27-5140–01), and 2 μg of footprints was obtained. Ribosomal RNA removal was performed using the Ribo-Zero Gold rRNA Removal Kit (Human/Mouse/Rat) (Illumina, MRZG12324). First, the footprints were excised from a Novex 15% TBE Urea PAGE gel (Thermo Fisher Scientific, EC68852BOX), and the 3′ ends were treated with T4 PNK (Biozym, P0503K) to enable ligation to a pre-adenylated linker. Subsequently, the RNA was reverse transcribed using Reverse Transcriptase (Biozym, ERT12925K), and the resulting cDNA was purified using a Novex 10% TBE Urea PAGE gel (Thermo Fisher Scientific, EC68752BOX). To circularize the fragments, Circligase I (Biozym, CL4115K) was employed, followed by PCR amplification and size selection using a Novex 8% TBE PAGE gel (Thermo Fisher Scientific, EC62152BOX). To ensure quality control, the size distributions of the ribosome profiling libraries were assessed using a Bioanalyzer 2100 (Agilent, 5067-1511) with a high-sensitivity DNA assay (Agilent, 5067–4626). The libraries were then multiplexed and sequenced on a NovaSeq 6000 Illumina platform, generating single-end 1 × 51-nucleotide reads.

### Stranded mRNA sequencing

To isolate polyA+ RNA, 3 million cells per iPSC and iPSC-CM sample, or 5–10 mg of snap-frozen and powdered tissue per adult heart sample, matching the identical samples previously processed for ribosome profiling, were treated with TRIzol reagent (Invitrogen, 15596018). The quality of the isolated RNA was assessed by measuring the RNA integrity number (RIN) using the RNA 6000 Nano assay on a Bioanalyzer 2100. High-quality RNA samples with an average RIN of 7.4 (tissue) and 9.7 (cells), respectively, were subjected to polyA purification to generate mRNA sequencing (mRNA-seq) libraries. The library preparation followed the TruSeq Stranded mRNA Reference Guide, using 1,000 ng of total RNA as input. Subsequently, the libraries were multiplexed and sequenced on an Illumina NovaSeq 6000 platform, generating paired-end 2 × 101-nucleotide reads.

### Generation of de novo transcript assemblies

First, we clipped Ribo-seq and RNA-seq datasets for residual adaptor sequences using cutadapt^68^. Ribo-seq datasets were additionally filtered for common contaminants: mitochondrial, rRNA and tRNA sequences.

To build a complete cardiac transcriptome of human, NHP and rodent species, we mapped the generated iPSC-CM and LV paired-end mRNA-seq datasets to the respective Ensembl genomes: GRCh38/hg38, Pan_tro_3.0/panTro5, gorGor4, Mmul_10/rheMac10, GRCm38/mm38 and Rnor6.0/rn6. Due to the absence of iPSC-CM datasets for mouse and rat, we additionally included the cardiac datasets from Cardoso-Moreira et al.^9^ for transcriptome assembly. We used the standard STAR (version 2.7.3a)^9^ settings with the following modified parameters: *–outSAMtype BAM SortedByCoordinate*, *–outFilterMismatchNmax 4*, *–outFilterMultimapNmax 20*, *–alignSJDBoverhangMin 6*, *–alignSJoverhangMin 500*, *–outFilterType BySJout*, *–limitOutSJcollapsed 10000000*, *–limitIObufferSize* = *300000000*and *–outFilterIntronMotifs RemoveNoncanonical*. We next included the XS tags with strand information to the generated BAM files, and we ran StringTie (version 2.1.1)^69^ with parameters *-M 0.5 -j 3 -f 0.2* to reconstruct de novo transcriptome for each sample, guided by a reference transcriptome annotation (Ensembl release 98 (ref. ^70^)). Genes were defined as independent loci composed of either annotated or assembled overlapping transcripts, regardless of the protein-coding status of the loci. We classified genes as ‘protein coding’, ‘lncRNA’ and ‘pseudogene’ based on defined gene biotypes in Ensembl, whereas the rest of genes not mapped to any Ensembl annotations were defined as ‘unannotated’. Later, all novel annotations were merged into species-specific consensus GTFs using StringTie merge, with parameters *-m 200 -F 0.5 -f 0.2* and cuffcompare. Transcripts assigned to codes ‘p’, ‘s’ and ‘c’ were excluded, and any unnanotated transcript with full overlap to an annotated CDS was re-annotated as protein coding.

### Dataset alignment to de novo transcript assemblies

We next remapped all Ribo-seq and RNA-seq datasets to the generated de novo transcript assemblies. The first read pair of each paired-end RNA-seq dataset was trimmed to 29 base pairs (bp) to process and map RNA-seq reads with the exact same parameters as the Ribo-seq data. We then ran STAR using similar settings as indicated before (see ‘Generation of de novo transcript assemblies’ subsection) but readjusting the maximum number of mismatches: *-outFilterMismatchNmax 2*. For subsequent analysis, we filtered out transcripts and genes with low RNA-seq expression values (fragments per kilobase of transcript per million mapped reads (FPKM) < 0.5 using pooled RNA-seq for iPSC-CMs and LVs).

### Detection of ORFs predicted by Ribo-seq

For each Ribo-seq sample, we searched for ORFs in each species transcriptome running ORFquant^71^ (version 1.00, cognate ORFs) and PRICE^72^ (version 1.0.3b, cognate and non-AUG ORFs), with standard settings and considering only uniquely mapped reads. Significant ORFs by sample in any of the two software (adjusted *P* < 0.05) were pooled into unique lists per species, collapsing in-frame ORFs where multiple instances of ORF isoforms shared ≥90% of the sequence, prioritizing cognate ORFs starting with AUG and selecting the longest ORF as representative. Later, each ORF was assigned to the most highly expressed transcript isoform based on the pooled RNA-seq FPKM levels calculated by StringTie. Any gene harboring at least one translated ORF or CDS was defined as translated.

The total numbers of pooled ORFs are displayed in Extended Data Fig. 4b. A high fraction of identified ORFs outside of CDS sequences (~61%) started with non-AUG initiation codons (Extended Data Fig. 4e) and exhibited similar characteristics compared to AUG cognate ORFs (Extended Data Fig. 4f,g).

### Defining sets of actively transcribed genes and replicated ORFs

To maximize the robustness of each inter-species comparison, we defined a set of ‘actively transcribed’ genes for each species based on their mean RNA-seq expression levels in iPSC-CMs and/or LV, measured in FPKM. Genes were included if they had a mean RNA-seq expression level of at least 1 FPKM and were expressed (FPKM ≥ 0.5) in at least three independent replicates. For pseudogenes and unannotated genes, only unique reads were considered for quantification. The total number of actively transcribed genes and their respective biotypes is represented in Fig. 1b.

In addition, we established a set of ‘replicated’ ORFs for each species by selecting ORFs that were partially or totally predicted in at least three samples and were encoded by actively transcribed genes. We then defined CDSs by selecting ORFs that overlapped in-frame annotated CDSs, considered canonical and assigned then to individual annotated genes when several were fused (Supplementary Data 1). The rest of cases were termed small ORFs (sORFs) because they are unannotated, and the vast majority (~91%) are shorter than 150 amino acids. The total numbers of replicated sORFs are illustrated in Fig. 5a. ORF biotypes were defined following a slightly modified version of the Phase I GENCODE nomenclature^18^ (Supplementary Data 2). Lastly, we used RiboseQC to extract P-site counts, which were employed for quantifying in-frame P-site counts associated with each ORF.

### Homology searches and identification of robust orthologs

We used the ‘pyliftover’ package (version 0.4.1) to identify counterpart sequences of exons from actively transcribed genes and replicated ORFs (sORFs and canonical CDSs) in the primate and rodent genomes considered in this study. Homologous regions were identified when a gene or ORF was fully mapped to unique counterpart regions on the same strand and chromosome in another species. Among the total CDSs as well as sORFs with young translation (see ‘Evolutionary classification of sORFs’ subsection), only one from humans (a young sORF), four from chimpanzees (two CDSs and two young sORFs) and 25 from rhesus (21 CDSs and four young sORFs) could not be aligned to the genomes of any other primate species (Extended Data Figs. 1e and 4c). Additionally, we assessed the mappability of 29-bp-long reads to identify ORF counterpart regions that could not be quantified due to the short length of Ribo-seq reads. Reassuringly, most ORFs exhibit fully mappable sequences (Extended Data Figs. 1e and 4c), with only 16 instances in humans (four CDSs and 12 young sORFs), 11 in chimpanzees (four CDSs and seven young sORFs) and 24 in rhesus (12 CDSs and 12 young sORFs) being unmappable in other analyzed primate species. These cases are described in Supplementary Table 9.

When the region was aligned to other species, we defined homologous genes and ORFs as those with the longest overlap to the identified homologous region, including all transcribed genes and all predicted ORFs in the other species to maximize homolog detection. In addition, we ran BLASTn^73^ (version 2.14.0+) against the same sequences of transcripts and ORFs per each species, identifying sequence homologs with a minimum E-value cutoff of 10^−4^. Genes or ORFs showing significant alignment to homologous sequences, as identified by pyliftover and/or BLAST, were considered as preserved in the respective species.

Next, we established two sets of robust orthologs in primates and mammals. This selection was based on identifying genes and ORFs with consistent homolog matches using both pyliftover and BLAST across all species considered in the lineage. Specifically, we included all genes and ORFs for which the same homolog match could be found as actively transcribed or with replicated translation in both pyliftover and BLAST analyses.

### Evolutionary classification of genes

We defined three evolutionary gene ages based on their presence or absence across species hearts. Genes identified uniquely in human, chimpanzee or rhesus macaque genomes that lacked detected homologs in other species were categorized as young and species specific. Additionally, genes exhibiting transcription unique to both humans and chimpanzees were defined as young and hominini specific. The transcription of the remaining genes was classified as preserved, forming a heterogeneous group with varying levels of conservation. It is important to note that we classified genes only for the three primate species in which we generated both iPSC-CM and LV data.

It should be noted that, due to the search for homologs being conducted by examining all transcribed genes in other species, certain hominini-specific genes may meet the expression thresholds in only one of the two species (human or chimpanzee). This discrepancy explains the difference in the number of identified hominini-specific genes between humans and chimpanzees.

### Evolutionary classification of sORFs

As done with the genes, we established three evolutionary sORF ages based on their presence or absence across species. The unique translation of sORFs identified in human, chimpanzee or rhesus macaque genomes that lacked detected homologs in other species and without evidence of translation in their counterpart sequences (fewer than 10 Ribo-seq reads in all pooled samples) was categorized as young and species specific. sORFs with translation unique to both humans and chimpanzees were defined as young and hominini specific. The remaining sORFs were defined as preserved at the level of translation.

Later, we employed a modified version of our previously published methodology^21^ to identify young sORFs with recent structures. For this, we reanalyzed the pairwise liftover alignments generated to trace the evolutionary origins of young sORFs. If the sequence of a sORF was truncated by at least 70% of the in-frame sequence in the counterpart regions of the other species analyzed, we classified the structure of the sORF as ‘de novo*’*. Otherwise, the structure of the sORF was defined as ‘intact’. For the cases where the region could not be aligned in other species, the mode of evolution could not be accurately assessed due to limited available data, because the ancestral region evolved at the same time as the ORF sequence. Consequently, these cases were classified as ‘orphan’.

### Analysis of differential gene and ORF expression

We defined sets of differentially transcribed and translated (based on RNA-seq counts or in-frame P-sites) genes and ORFs using DESeq2 (version 1.26.0)^74^.

For the calculation of differential TE, we evaluated the differences in ribosome occupancies (in-frame P-sites) between each primate species and the two other primates with DESeq2 and used RNA-seq counts as an interaction term into the statistical model of DESeq2.

Genes and sORFs that were uniquely differentially transcribed or translated in iPSC-CMs or prenatal LVs were defined as ‘prenatal’, whereas genes and sORFs that were uniquely differentially transcribed or translated in adult or postnatal LVs were defined as ‘postnatal’.

### Calculation of TEs and their variances

TE was calculated as the ratio of DESeq2-normalized in-frame Ribo-seq P-sites and total RNA-seq counts in LV samples. Specifically, these ratios were computed for the primary CDS of each gene. We focused on cases where the CDS and its corresponding host gene were identified as robust orthologs within the mammalian (4,524 genes) or primate (8,238 genes) lineage. Most of the aligned CDS homologs displayed similar lengths across primate species (Extended Data Fig. 1f). However, in instances where multiple CDS sequences were encoded by a single gene, we selected the CDS region with the lowest variance in nucleotide length across species. Raw counts were based on in-frame P-sites and RNA-seq counts and adjusted by the CDS length to ensure comparability across different species prior to performing normalization across all samples and species. Genes that exhibited median values of 0 or 3 or more 0 count values across replicates within a specific species were excluded from global TE calculations.

We also determined the TE_var_ among five species (human, chimpanzee, rhesus, mouse and rat) for the aforementioned gene sets. To achieve this, we employed a multi-step approach. Initially, we computed the inter-species variances of the estimated individual TE for each species. This involved calculating the variance of TE estimates across all individuals in each species and subsequently dividing them by the number of samples. By averaging these normalized inter-species variances across the five species, we derived the average inter-species variance. Later, we evaluated the intra-species variance of average TE values for the five species. Subsequently, we subtracted inter-species variances from each calculated intra-species variance value. This resulted in a TE_var_ score for each gene under consideration. Notably, a higher gene TE_var_ score implies higher level of inter-species variability in TE corrected by each intra-species variance.

To assess potential effects resulting from species alterations in gene structure within TE_var_, we computed variances in nucleotide length for both the coding sequence (CDS_var_) and its associated untranslated region (UTR_var_) across the five species. We later divided the genes in four groups corresponding to the four quantiles of CDS_var_ and UTR_var_. To evaluate the correlation between TE_var_ and the levels of natural selection acting on the CDSs, we downloaded pre-computed ratios of non-synonymous to synonymous substitutions (dN/dS) from Ensembl^70^.

### Gene markers and sORFs evaluated in CRISPR–Cas9 screens

We curated different lists of genes and sORFs in our study:

- A list of cardiac gene markers based on mining several published publications (Supplementary Table 10). These genes represent cardiac maturity markers, sarcomere components and cardiac-specific genes.
- OXPHOS components were extracted based on Gene Ontology annotations^75^.
- Cardiac cell type markers from the Human Heart Cell Atlas, retrieving all genes that showed more than 90% of total cardiac expression restricted to one cell type^76,77^.
- Essential genes were retrieved from a list of proteins that were shown to be essential for survival of human cells in a previous study^27^. By doing mutagenesis in haploid human cells, the authors identified genes required for optimal fitness. We only included genes that affected cell viability in both tested cell lines (KMB7 and HAP1).
- Four sORF lists from CRISPR–Cas9 screens evaluating cell survival in human cell lines^37–40^.

### Calculation of tissue enrichments

Tissue enrichment indexes were based on the tau (τ) metric^78^:

$$\tau =\mathop{\sum }\limits_{i=1}^{n}\frac{(1-{{xt}}_{i})}{n-1}{{;}}\quad{{xt}}_{i}=\frac{{x}_{i}}{{\max }_{1 < i < n({x}_{i})^{\prime} }}$$

where *n* is the number of tissues and *x*_*i*_ is the expression of a gene/ORF in a tissue.

For the calculation of the tissue enrichment, we used mean expression values across each tissue for (1) the developmental resource of Cardoso-Moreira et al.^9^ and (2) the GTEx project^34^. Owing to the known pervasive expression of a high number of evolutionarily young genes in the testis^8,25^, we excluded this tissue from tissue enrichment calculations. Any gene and ORF with τ above 0.75 were considered as tissue enriched.

Finally, we defined a list of genes with recent cardiac-enriched expression (unique to humans) by selecting cases with τ_heart_ above 0.75 uniquely in humans.

### Genome-wide gene–gene correlations

Spearman correlations were computed to assess co-regulation among all transcribed genes in both human and rhesus, as we did previously^15,79^. DESeq2-normalized counts were used, focusing on pairwise complete observations across cardiac developmental RNA-seq datasets^9^. We required a minimum of 20 (human) or 10 (rhesus) samples with at least one raw count to be included in the calculation. Subsequently, for each young gene, we aggregated all co-regulated protein-coding genes (with an absolute Spearman’s rho > 0.5) and searched for enriched functionalities (see ‘Functional enrichment analysis’ subsection).

### Analysis of ClinVar genetic variants

We evaluated genetic variants that can be important for disease in ClinVar^45^ (database March 2024) by quantifying how many of the genes and sORFs of interest overlapped at least one germline variant classified as pathogenic or likely pathogenic. Variants involved in cardiomyopathy, atrial fibrillation, ventricular tachycardia or cardiovascular phenotype were considered as cardiac disease variants.

### Prediction of motifs and stable structures in microproteins

We predicted motifs and stable structures of microprotein sequences encoded by sORFs using InterProScan^80^ (version 5.69-101.0) and ESMFold^81^ (version 1). In parallel, we retrieved motifs and structures of shuffled amino acid sequences of all microproteins encoded by sORFs. Any structure with an average pLDDT higher than 80 was defined as potentially well folded. Due to limitations in ESMFold, only sORFs shorter than 400 amino acids were predicted.

### CRISPRi-mediated knockdown with single-cell RNA-seq output

We focused on two candidate genes: *LINC01405* and *SRP14-AS1*. These are two examples of cardiac genes where both the gene and the encoded sORF emerged recently in primates; their expression is upregulated in iPSC-CMs compared to adult LVs; and they are dysregulated in DCM. See Supplementary Data 4 for a full description of the method.

### Functional enrichment analysis

Functional Gene Ontology^75^, KEGG^82^ and CORUM^83^ enrichment on specific groups of genes was done with gProfiler2 (ref. ^84^)(version 0.2.0), with default parameters and selecting full sets of transcribed or translated genes as custom backgrounds based on selected cutoffs for the different analyses, such as expression level, replicability or robust conservation.

### Statistics and reproducibility

Statistical analysis was performed using R (http://r-project.org).

Differentially expressed and translated genes were identified with DESeq2. Cases with an absolute fold change equal to or higher than 1.5 and an adjusted *P* value lower than 0.05 were defined as differentially expressed/translated. For differential gene expression analysis in single-cell RNA-seq libraries, we used Wilcoxon rank-sum test considering differentially expressed genes with false discovery rate (FDR) < 0.05 and log_2_ fold change > 0.5. Comparisons between proportions of genes were analyzed with two-sided Fisherʼs test, unless otherwise stated. *P* < 0.05 was considered significant, and *P* values were adjusted using FDR corrections for several tests. Comparison between groups of ORFs in Extended Data Fig. 4k was evaluated by Wilcoxon rank-sum test; *P* < 0.05 was considered significant. Comparisons between multiple groups in Fig. 1c were made by ANOVA; *P* < 0.05 was considered significant. For Fig. 5l and Extended Data Fig. 4m, we compared whether microproteins encoded by sORFs presented a significantly higher proportion of motifs and stable structures compared to shuffled sequences by running one-sided Fisher’s exact tests; *P* < 0.05 was considered significant, and *P* values were adjusted using FDR corrections for several tests.

We evaluated the significance of the calculated TE_var_ observations using two approaches displayed in Extended Data Fig. 1g: (1) resampling, randomly assigning each of the analyzed samples to each species and recalculating the TE_var_ in each iteration (10,000 iterations) and (2) data downsampling, by selecting three (*n* = 10,000 iterations) and four (this is the lowest number of samples in a considered species, *n* = 10,000 iterations) samples per species to recalculate TE_var_ and evaluate the robustness of the calculated TE_var_ in our original data after modifying the number of samples per species. Both approaches were applied without sample replacement. One-sided *P* values obtained using both approaches represent the fraction of resampled or downsampled observations (out of 10,000) with medians higher than the real observed values and were adjusted using FDR corrections for the five groups of tested genes (essential, cardiac-enriched, sarcomere, OXPHOS complex IV and OXPHOS complex V). Resampling analyses indicated that the observed high TE_var_ of OXPHOS complex IV subunits was borderline significant (*P* = 0.047), whereas the TE_var_ of OXPHOS complex V subunits showed higher significance (*P* = 0.004). Further downsampling of the dataset over 10,000 iterations, including the same number of individuals per species, confirmed that the observed high TE_var_ for both complexes was significantly robust to differences in the number of biological replicates per species, although these values exhibited high variability when the number of samples was lower (complex IV downsampled to three samples: 95% confidence interval (CI_95%_) (0.51, 1.56); complex IV downsampled to four samples: CI_95%_ (0.64, 1.13); complex V downsampled to three samples: CI_95%_ (0.93, 1.83); and complex V downsampled to four samples: CI_95%_ (1.04, 1.52)).

Sample numbers are indicated in the figure panels or legends. Reproducibility was ensured by having a minimum of three biological replicates per species and tissue. For iPSC-CMs, we generated three differentiation replicates (five for human) of the same individual. No statistical method was used to pre-determine sample size, and no data were excluded from analysis.

### Reporting summary

Further information on research design is available in the Nature Portfolio Reporting Summary linked to this article.

## Supplementary information

Supplementary Data 1–4 Supplementary Data 1: Evaluation of fusion genes in primate assemblies. Supplementary Data 2: Definition of ORF biotypes. Supplementary Data 3: Figure exemplifying the gating strategy for flow cytometry. Supplemetary Data 4: CRISPRi-mediated knockdown with single-cell RNA-seq output.

Reporting Summary

SSupplementary Tables 1–10 Supplementary Table 1: Sample description. Supplementary Table 2: Human young genes. Supplementary Table 3: Chimpanzee young genes. Supplementary Table 4: Rhesus macaque young genes. Supplementary Table 5: Information of 76 conserved genes with human-specific cardiac enrichment. Supplementary Table 6: Human, chimpanzee and rhesus young sORFs. Supplementary Table 7: Mapped ClinVar variants to young genes and sORFs. Supplementary Table 8: Information of antibodies. Supplementary Table 9: List of unaligned and unmappable CDSs and ORFs. Supplementary Table 10: Description of selected cardiac markers.

## Source data

Source Data Fig. 1 Statistical source data.

Source Data Fig. 2 Statistical source data.

Source Data Fig. 3 Statistical source data.

Source Data Fig. 4 Statistical source data.

Source Data Fig. 5 Statistical source data.

Source Data Fig. 6 Statistical source data.

Source Data Extended Data Fig. 1 Statistical source data.

Source Data Extended Data Fig. 2 Statistical source data.

Source Data Extended Data Fig. 3 Statistical source data.

Source Data Extended Data Fig. 4 Statistical source data.

Source Data Extended Data Fig. 5 Statistical source data.

## Data Availability

The source data generated during the current study are included in the main article as well as in supplementary tables. All Ribo-seq and RNA-seq data created in this study have been deposited online and are publicly available as of the date of publication in the European Nucleotide Archive (ENA) under accession code PRJEB65856. For completeness, we retrieved matched Ribo-seq and RNA-seq datasets corresponding to human left ventricle (*n* = 15 controls and *n* = 65 end-stage DCM samples, European Genome-phenome Archive (EGA) EGAS00001003263)^15^; rat left ventricle (*n* = 5, ENA accession code PRJEB29208)^15^; mouse left ventricle (*n* = 6, ENA accession code PRJEB29208)^15^; human brain (*n* = 3, ArrayExpress accession code E-MTAB-7247)^26^; rhesus brain (*n* = 3, ArrayExpress accession code E-MTAB-7247)^26^; and mouse brain (*n* = 3, ArrayExpress accession code E-MTAB-7247)^26^. Additionally, we downloaded RNA-seq datasets corresponding to additional human left ventricles (*n* = 97 controls and *n* = 108 dilated cardiomyopathy samples, EGA EGAS00001002454)^44^; human myocardial samples (*n* = 9 controls and *n* = 28 hypertrophic cardiomyopathy samples, National Center for Biotechnology Information Sequence Read Archive code SRP186138)^42^; as well as different stages of organ development for human (*n* = 363, ArrayExpress accession code E-MTAB-6814)^9^, rhesus macaque (*n* = 177, ArrayExpress accession code E-MTAB-6813)^9^, rat (*n* = 362, ArrayExpress accession code E-MTAB-6811)^9^ and mouse (*n* = 317, ArrayExpress accession code E-MTAB-6798)^9^. All annotation GTF and genome FASTA files were retrieved from Ensembl^70^. Multiple alignment chain files were retrieved from the UCSC Genome Browser database^85^.

## Code availability

Custom code for bioinformatic analyses and a full list of predicted coding sequences are available through GitHub at https://github.com/jorruior/RuizOrera_etal_2024.
