# Supplementary material for: Evolution of translational control and the emergence of genes and open reading frames in human and non-human primate hearts
Source: Nat Cardiovasc Res. 2024 Sep 24;3(10):1217–35. doi: 10.1038/s44161-024-00544-7 (PMC11473369; doi:10.1038/s44161-024-00544-7)
Supplement: Supplementary file 1 — Supplementary Data 1: Evaluation of fusion genes in primate assemblies. Supplementary Data 2: Definition of ORF biotypes. Supplementary Data 3: Figure exemplifying the gating strategy for flow cytometry. Supplemetary Data 4: CRISPRi-mediated knockdown with single-cell RNA-seq output. [file 44161_2024_544_MOESM1_ESM.pdf]

nature cardiovascular research

Supplementary information https://doi.org/10.1038/s44161-024-00544-7

Evolution of translational control and the emergence of genes and open reading frames in human and non-human primate hearts

In the format provided by the authors and unedited

Evolution of translational control and the emergence of genes and open reading frames in human and non-human primate hearts

Supplementary Data 1. Evaluation of fusion genes in primate assemblies.

Due to the presence of isoforms overlapping multiple annotated genes, 948, 514, and 606 human, chimp, and rhesus loci contained several merged annotated protein-coding genes. However, Ribo-seq allows the detection of multiple CDSs per assembled loci -this also includes merged annotated genes- when supported by significant three-frame periodicity. Therefore, we distinguished independent predicted CDS regions inside merged genes with Ribo-seq. Specifically, we assigned each predicted CDS to a unique annotated protein-coding gene (based on Ensembl CDS annotations) when the predicted and the annotated sequences were identical. For example, rhesus *MYH6* and *MYH7* were assembled as a single locus, and both genes are also annotated as a single locus in rhesus in Ensembl v.98 despite having independent CDS annotations for *MYH6* and *MYH7* proteins. Rhesus fusion *MYH6-MYH7* gene had two non-overlapping CDSs that we could unambiguously map to Ensembl CDSs *MYH6* (orf_id: P1_7:85423577_85448357:-:37:5820) and *MYH7* (orf_id: P1_7:85453986_85474693:-:38:5808), enabling the quantification of their RNA-seq and RIbo-seq levels independently. Hence, TE calculations could be done individually for *MYH6* and *MYH7* as well as for other protein-coding genes that underwent assembly fusion.

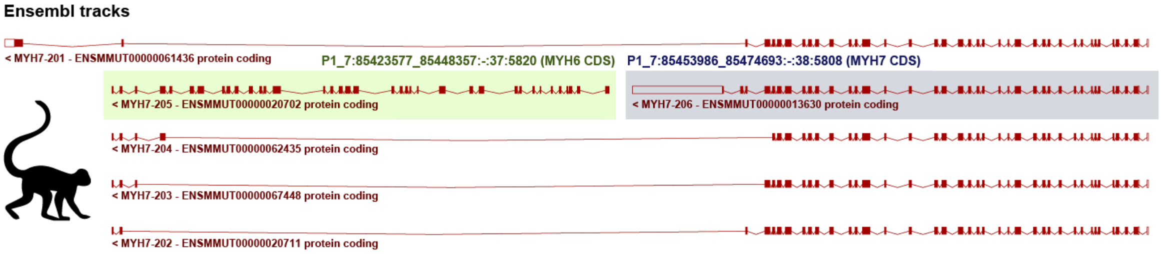

Supplementary Data 2. Definition of ORF biotypes.

- Pseudogene: sORFs encoded by genes overlapping annotated pseudogenes.
- LncRNA-ORFs: sORFs encoded by presumed long non-coding RNAs and unannotated genes.
- NcRNA-ORFs: sORFs encoded by non-coding or unannotated isoforms from protein-coding genes.
- Upstream ORFs or uORFs: encoded by 5’ UTR sequences.
- Upstream overlapping ORFs or uoORFs: encoded by 5’ UTR sequences and partially overlapping an annotated CDS in an alternative frame.
- Internal ORFs or intORFs: fully overlapping an annotated CDS in an alternative frame.
- Downstream ORFs or dORFs: encoded by 3’ UTR sequences.
- Downstream overlapping ORFs or doORFs: encoded by 3’ UTR sequences and partially overlapping an annotated CDS in an alternative frame.

Supplementary Data 3. Figure exemplifying the gating strategy for flow cytometry in chimpanzee.

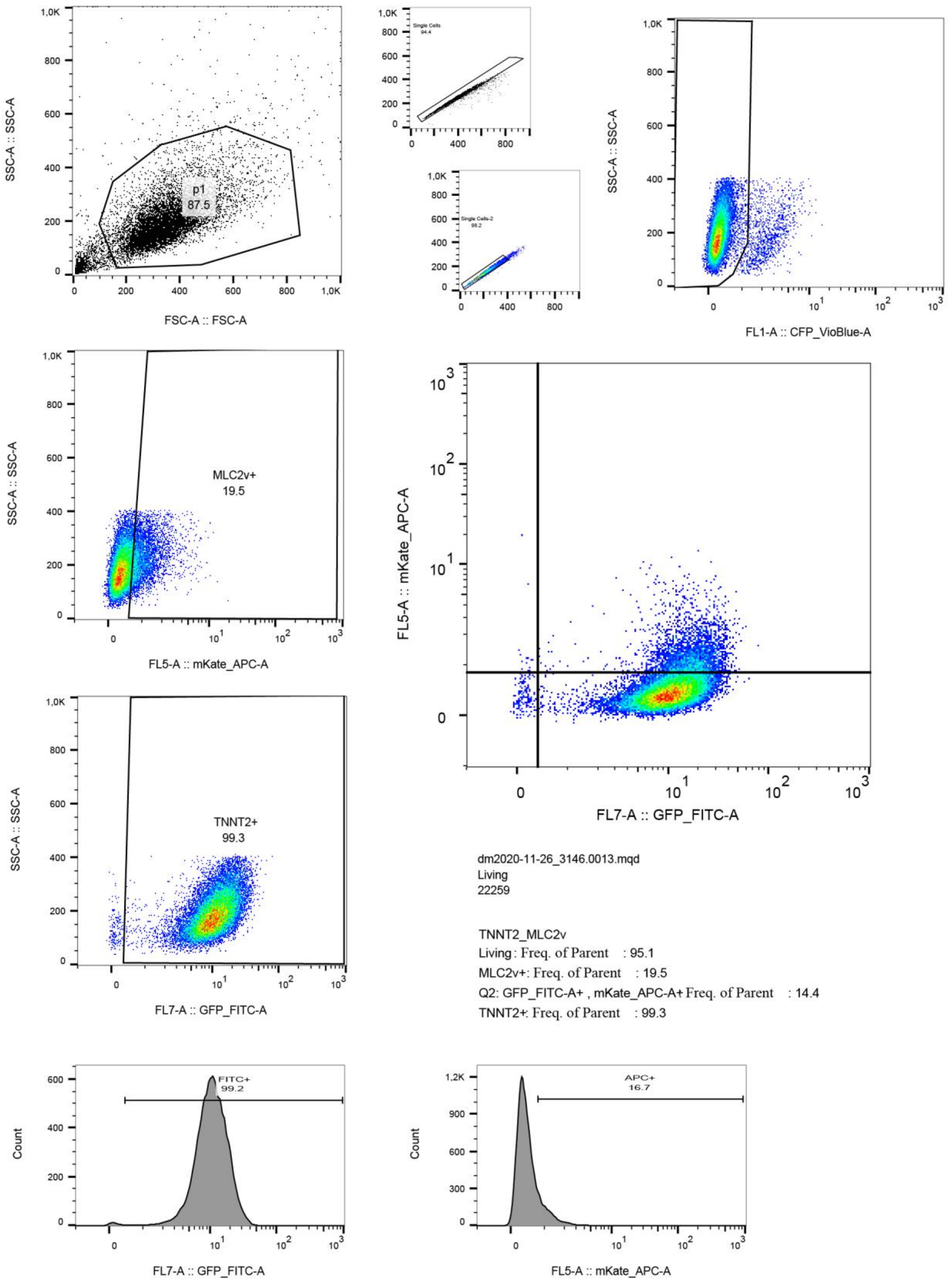

Supplementary Data 4. CRISPRi-mediated knockdown of LINC01405 and SRP14-AS1 with single cell RNA-seq output

To perturb the expression of *LINC01405* and *SRP14-AS1* in iPSC-CMs, CRISPR interference (CRISPRi) sgRNA sequences were designed using CRISPick (Broad Institute). The five top-scoring sgRNAs per gene were chosen:

Target.Gene.ID,Target.Gene.Symbol,TSS.Position,PAM.Policy,Strand.of.sgRNA,Orientation,sgRNA.'Cut'.Position,sgRNA.Sequence,sgRNA.Context.Sequence,PAM.Sequence,sgRNA.'Cut'.Site.TSS.Offset

ENSG00000185847,LINC01405,110936577,SpyoCas9,-,antisense,110936579,GAGAGACAGAGAGAGAAGTG,GCAGGAGAGACAGAGAGAGAAGTGAGGAGA,AGG,2

ENSG00000185847,LINC01405,110936577,SpyoCas9,-,antisense,110936662,CAGTATGACTGGGAGACCTC,CCAGCAGTATGACTGGGAGACCTCAGGAAA,AGG,85

ENSG00000185847,LINC01405,110936577,SpyoCas9,-,antisense,110936672,GCTTAACCAGCAGTATGACT,GCAGGCTTAACCAGCAGTATGACTGGGAGA,GGG,95

ENSG00000185847,LINC01405,110936577,SpyoCas9,-,antisense,110936673,GGCTTAACCAGCAGTATGAC,TGCAGGCTTAACCAGCAGTATGACTGGGAG,TGG,96

ENSG00000185847,LINC01405,110936577,SpyoCas9,+,sense,110936677,AGGTCTCCCAGTCATACTGC,CCTGAGGTCTCCCAGTCATACTGCTGGTTA,TGG,100

ENSG00000248508,SRP14-AS1,40039320,SpyoCas9,-,antisense,40039323,AGGACAGCCCCAGCGCCTCC,ACCCAGGACAGCCCCAGCGCCTCCGGGTCA,GGG,3

ENSG00000248508,SRP14-AS1,40039320,SpyoCas9,-,antisense,40039343,TTTCTCGCATGTGACGACCC,ACGATTTCTCGCATGTGACGACCCAGGACA,AGG,23

ENSG00000248508,SRP14-AS1,40039320,SpyoCas9,-,antisense,40039374,TCACTCCGTCACACTGGCTG,GCTCTCACTCCGTCACACTGGCTGGGGTGA,GGG,54

ENSG00000248508,SRP14-AS1,40039320,SpyoCas9,-,antisense,40039375,CTCACTCCGTCACACTGGCT,TGCTCTCACTCCGTCACACTGGCTGGGGTG,GGG,55

ENSG00000248508,SRP14-AS1,40039320,SpyoCas9,+,sense,40039327,CCTGTGACCCGGAGGCGCTG,GGGGCCTGTGACCCGGAGGCGCTGGGGCTG,GGG,7

In addition, a subset of scrambled negative control sgRNAs[1] were added. To allow for enrichment of cells that obtained a sgRNA with antibiotics or FACS, PuroR cassette from CROPseq-Guide-Puro (Addgene #86708)[2] was replaced with a *BSD* (Blasticidin S deaminase)-P2A-iRFP670 cassette. Subsequently, sgRNA sequences were inserted, abundance of all the designed sgRNAs was validated with next-generation sequencing and and 3rd generation lentiviruses were produced by transfecting HEK293T cells with the plasmid library, and pMDLg/pRRE (Addgene 12251), pRSV-Rev (Addgene 12253), and pMD2.G (Addgene 12259) packaging plasmids. Lentivirus-containing medium was collected 24 h and 48 h after transfection. Lentivirus titers were measured using Lenti-X qRT-PCR Titration Kit (Takara).

RUCDRi002-A-17 (CRISPRi2) human iPSCs stably expressing a dCas9-KRAB-tdTomato cassette[3] were cultivated in E8 (Thermo) on plates coated with Matrigel (Corning). Every 3-4 days, cells were enzymatically dissociated with Accutase (Thermo) and replated in E8 containing 10 µM Rho-kinase inhibitor Y-27632. Twenty-four hours after replating, iPSCs were transduced with a MOI of 1, transduced iPSCs were enriched by FACS three days after transduction. Differentiation to mesoderm was initiated 7-9 days after sorting. Four differentiation experiments were carried out (see **iPSC-cardiomyocyte differentiation** in Methods). At day 30, iPSC-CMs were dissociated using 10x TrypLE (Thermo), resuspended in PBS + 2 % BSA and automatically counted using a Countess III (Life Technologies). Cell suspension was loaded on the Chromium Controller (10X Genomics) with targeted cell recovery of 10,000 per reaction.

3′ gene expression libraries were prepared according to the manufacturer's instructions for the v3 Chromium Single Cell Reagent Kits (10X Genomics). Quality of the cDNA library was determined using TapeStation High Sensitivity D5000 (Agilent) and KAPA Library Quantification kit (Illumina). 10X libraries were sequenced on Illumina NovaSeq with a targeted read number of 50,000 reads per cell.

For amplicon sgRNA PCRs, 10 ng of 10X cDNA were used as starting material and each round of PCR amplification with tprimers described in Alda-Catalinas *et al.*, 2020[4] was monitored by KAPA SYBR FAST (Illumina), reactions were aborted after reaching inflection point. PCR products were purified with 1X AMPure XP (Beckman Coulter) cleanup. One microliter of a 1:5 dilution of the first PCR product and one microliter of a 1:25 dilution of the 2nd PCR product were taken into the following reactions. 6 reactions were performed per sample to compensate for PCR bias. Quality of the amplicon sgRNA libraries was determined using TapeStation High Sensitivity D5000 (Agilent) and KAPA Library Quantification kit (Illumina). Amplicon sgRNA libraries were sequenced on Illumina NextSeq 500.

10X scRNA-seq data were processed using standard CellRanger (v.3.1.2) for mapping to the human reference human genome with a modified pre-mRNA Ensembl gtf file. The concatenated filtered feature-barcode matrices were analyzed with Scanpy[5] (v1.5.1). Single cells were filtered for counts (5,000 ≤ n_counts), genes (3,000 ≤ n_genes ≤ 12,000), mitochondrial genes (percent_mito ≤ 25%), and ribosomal genes (percent_ribo ≥1%).

For differentially expressed gene analysis after knockdown of *LINC01405* and *SRP14-AS1*, read counts were normalized and logarithmically transformed. Effects of percentage of

mitochondrial genes and total counts per cell were regressed out and values were scaled to unit variance. Potential batch effects were studied by calculating principal components, selected principal components were harmonised samplewise by using Harmonypy[6] (v0.0.4). Cells were clustered using the network-based Louvain and Leiden algorithms, such that each cluster consisted of cells from all samples with non-significantly different composition.

From each amplicon read 1, the 10x barcode and the UMI were extracted. From each amplicon read 2, potential sgRNA sequence was extracted, reads were forwarded that exactly matched the designed gRNAs. Cell barcodes detected in the amplicon libraries were matched with barcodes detected in the regular 10X scRNA-seq libraries. Only sgRNAs detection events that accounted for ≥ 25% of the total gRNA-specific UMIs were considered. For *LINC01405* and *SRP14-AS1*, the population of cells with a sgRNA and the knockdown was carried forward to perform differential gene expression analysis against the cells that express both negative control sgRNAs and the targeted gene of interest using the Scanpy-implemented Wilcoxon rank-sum-test.

REFERENCES

1. Sanson, K. R. *et al.* Optimized libraries for CRISPR-Cas9 genetic screens with multiple modalities. *Nat. Commun.* **9**, 1–15 (2018).
2. Datlinger, P. *et al.* Pooled CRISPR screening with single-cell transcriptome readout. *Nat. Methods* **14**, 297–301 (2017).
3. Schoger, E., Zimmermann, W.-H., Cyganek, L. & Zelarayán, L. C. Establishment of two homozygous CRISPR interference (CRISPRi) knock-in human induced pluripotent stem cell (hiPSC) lines for titratable endogenous gene repression. *Stem Cell Res.* **55**, 102473 (2021).
4. Alda-Catalinas, C. *et al.* A Single-Cell Transcriptomics CRISPR-Activation Screen Identifies Epigenetic Regulators of the Zygotic Genome Activation Program. *Cell systems* **11**, (2020).
5. Wolf, F. A., Angerer, P. & Theis, F. J. SCANPY: large-scale single-cell gene expression data analysis. *Genome Biol.* **19**, 15 (2018).
6. Korsunsky, I. *et al.* Fast, sensitive and accurate integration of single-cell data with Harmony. *Nat. Methods* **16**, 1289–1296 (2019).
