# Supplementary material for: Evolution of translational control and the emergence of genes and open reading frames in human and non-human primate hearts
Source: Nat Cardiovasc Res. 2024 Sep 24;3(10):1217–35. doi: 10.1038/s44161-024-00544-7 (PMC11473369; doi:10.1038/s44161-024-00544-7)
Supplement: Supplementary file 2 — Reporting Summary [file 44161_2024_544_MOESM2_ESM.pdf]

nature portfolio

Corresponding author(s): Norbert Hübner, Jorge Ruiz-Orera

Last updated by author(s): Aug 23, 2024

Reporting Summary

Nature Portfolio wishes to improve the reproducibility of the work that we publish. This form provides structure for consistency and transparency in reporting. For further information on Nature Portfolio policies, see our Editorial Policies and the Editorial Policy Checklist.

Statistics

For all statistical analyses, confirm that the following items are present in the figure legend, table legend, main text, or Methods section.

n/a	Confirmed	
☐	☒	The exact sample size (*n*) for each experimental group/condition, given as a discrete number and unit of measurement
☐	☒	A statement on whether measurements were taken from distinct samples or whether the same sample was measured repeatedly
☐	☒	The statistical test(s) used AND whether they are one- or two-sided<br>*Only common tests should be described solely by name; describe more complex techniques in the Methods section.*
☒	☐	A description of all covariates tested
☐	☒	A description of any assumptions or corrections, such as tests of normality and adjustment for multiple comparisons
☐	☒	A full description of the statistical parameters including central tendency (e.g. means) or other basic estimates (e.g. regression coefficient) AND variation (e.g. standard deviation) or associated estimates of uncertainty (e.g. confidence intervals)
☐	☒	For null hypothesis testing, the test statistic (e.g. *F*, *t*, *r*) with confidence intervals, effect sizes, degrees of freedom and *P* value noted<br>*Give P values as exact values whenever suitable.*
☒	☐	For Bayesian analysis, information on the choice of priors and Markov chain Monte Carlo settings
☒	☐	For hierarchical and complex designs, identification of the appropriate level for tests and full reporting of outcomes
☒	☐	Estimates of effect sizes (e.g. Cohen's *d*, Pearson's *r*), indicating how they were calculated

Our web collection on statistics for biologists contains articles on many of the points above.

Software and code

Policy information about availability of computer code

Data collection	No specific software or code was used to collect the data in this manuscript.
Data analysis	Custom code for bioinformatic analyses is available through GitHub at https://github.com/jorruior/RuizOrera_etal_2024. In addition, these published software were used in the manuscript: STAR (v2.7.3a), Stringtie (v.2.1.1), ORFquant (v1.0), PRICE (v1.0.3b), pyliftover (v.0.4.1), BLAST (v2.14.0+), DESeq2 (v1.26.0), Interproscan (v5.69-101.0), ESMFold (v1), CellRanger (v.3.1.2), Scanpy (v1.5.1), Harmonypy (v0.0.4), gProfiler (v0.2.0)

For manuscripts utilizing custom algorithms or software that are central to the research but not yet described in published literature, software must be made available to editors and reviewers. We strongly encourage code deposition in a community repository (e.g. GitHub). See the Nature Portfolio guidelines for submitting code & software for further information.

Data

Policy information about availability of data

All manuscripts must include a data availability statement. This statement should provide the following information, where applicable:

- Accession codes, unique identifiers, or web links for publicly available datasets
- A description of any restrictions on data availability
- For clinical datasets or third party data, please ensure that the statement adheres to our policy

The source data generated during the current study are included in the main article as well as in Supplementary Tables. All Ribo-seq and RNA-seq data created in

this study have been deposited online and are publicly available as of the date of publication in the European Nucleotide Archive (ENA) accession code PRJEB65856. For completeness, we retrieved matched Ribo-seq and RNA-seq datasets corresponding to human left ventricle (n = 15 controls and 65 end-stage DCM samples, European Genome-phenome Archive (EGA) EGAS00001003263), rat left ventricle (n = 5, ENA accession code PRJEB29208), mouse left ventricle (n = 6, ENA accession code PRJEB29208), human brain (n = 3, ArrayExpress accession code E-MTAB-7247), rhesus brain (n = 3, ArrayExpress accession code E-MTAB-7247), and mouse brain (n = 3, ArrayExpress accession code E-MTAB-7247). Additionally, we downloaded RNA-seq datasets corresponding to additional human left ventricles (n = 97 controls and 108 dilated cardiomyopathy samples, EGA EGAS00001002454), human myocardial samples (n = 9 controls and 28 hypertrophic cardiomyopathy samples, NCBI Sequence Read Archive database SRP186138), as well as different stages of organ development for human (n = 363, ArrayExpress accession code E-MTAB-6814), rhesus macaque (n = 177, ArrayExpress accession code E-MTAB-6813), rat (n = 362, ArrayExpress accession code E-MTAB-6811)9 and mouse (n = 317, ArrayExpress accession code E-MTAB-6798). All annotation GTF and FASTA files were retrieved from Ensembl. Multiple alignment chain files were retrieved from UCSC.

Research involving human participants, their data, or biological material

Policy information about studies with human participants or human data. See also policy information about sex, gender (identity/presentation), and sexual orientation and race, ethnicity and racism.

Reporting on sex and gender	N/A
Reporting on race, ethnicity, or other socially relevant groupings	N/A
Population characteristics	N/A
Recruitment	N/A
Ethics oversight	N/A

Note that full information on the approval of the study protocol must also be provided in the manuscript.

Field-specific reporting

Please select the one below that is the best fit for your research. If you are not sure, read the appropriate sections before making your selection.

☒ Life sciences ☐ Behavioural & social sciences ☐ Ecological, evolutionary & environmental sciences

For a reference copy of the document with all sections, see nature.com/documents/nr-reporting-summary-flat.pdf

Life sciences study design

All studies must disclose on these points even when the disclosure is negative.

Sample size	No statistical method was used to predetermine sample size. For each of the sample collections (iPSC-CM, adult left ventricles) we generated at least three replicates per species, as n = 3 is the minimum required for any inferential analysis of population. Achieving larger sample sizes would have been challenging due to the difficulty of obtaining access to non-human primate samples.
Data exclusions	No data was excluded. The results of MARCHF11-AS1 knowckdown by CRISPRi were not included since the guide RNAs did not perform properly for this gene and the knockdown did not work out technically.
Replication	For non-human primate iPSC-CMs, we generated three differentiation rounds using reprogrammed fibroblasts from the same individual. We generated five differentiation rounds for human iPSC-CMs.<br>For primate left ventricles, we generated five (chimpanzee) and four (rhesus) biological replicates from different animals and processed the samples in two batches.<br>We generated principal component analysis (PCA), correlation plots, and comparison with publicly available sources to confirm that the replicates were correct, clustered together, and there were no batch effects or clear outliers.
Randomization	We checked the significance of our results on Translational Efficiency variances (TEvar) using two randomization methods. First, we used resampling, where we randomly shuffled the samples among species and recalculated the TEvar 10,000 times. Second, we used downsampling, where we selected three or four samples per species (based on the smallest group size) and recalculated the TEvar 10,000 times to test how changing the sample size affects the results.
Blinding	Given the computational nature of this study, we did not consider blinding to be relevant. Of note, data analysis was done independently of the samples and data used in these study.

Reporting for specific materials, systems and methods

We require information from authors about some types of materials, experimental systems and methods used in many studies. Here, indicate whether each material, system or method listed is relevant to your study. If you are not sure if a list item applies to your research, read the appropriate section before selecting a response.

Materials & experimental systems

n/a	Involved in the study	
☐	☒	Antibodies
☐	☒	Eukaryotic cell lines
☒	☐	Palaeontology and archaeology
☐	☒	Animals and other organisms
☒	☐	Clinical data
☒	☐	Dual use research of concern
☒	☐	Plants

Methods

n/a	Involved in the study	
☒	☐	ChIP-seq
☐	☒	Flow cytometry
☒	☐	MRI-based neuroimaging

Antibodies

Antibodies used

Antibody (Ig / -conjugate) Dilution Manufacturer Cat
POU5F1 (rabbit IgG) 0.111111111 Santa Cruz sc-9081
SSEA4 (mouse IgG) 0.111111111 Cell Signaling 4755
TRA-1-60 (mouse IgM) 0.111111111 Cell Signaling 4746
NKX2-5 (goat IgG) 0.111111111 R&D AF2444-SP
TNNT2 (mouse IgG) 0.111111111 Abcam AB10214
ACTN2 (mouse IgG) 0.180555556 Sigma A7811
MYL2 (rabbit IgG) 0.111111111 Proteintech 10906-1-AP
donkey anti-rabbit IgG AF555 0.388888889 Thermo A21206
donkey anti-mouse IgG AF488 0.388888889 Thermo A21202
goat anti-mouse IgM AF488 0.388888889 Thermo A21042
donkey anti-goat IgG AF555 0.388888889 Thermo A11055
TNNT2-FITC 01:50 Miltenyi 130-119-575
MYL2-APC 01:50 Miltenyi 130-106-134
REA-FITC (isotype control) 01:50 Miltenyi 130-113-449
REA-APC (isotype control) 01:50 Miltenyi 130-113-446

Validation

POU5F1 (rabbit IgG) Expected structural labelling (nuclear); validated and cited in a very broad range of mammals (manufacturer's website)
SSEA4 (mouse IgG) Expected structural labelling (cell surface); validated and cited in human and chimpanzee (manufacturer's website)
TRA-1-60 (mouse IgM) Expected structural labelling (cell surface); validated and cited in human (manufacturer's website)
NKX2-5 (goat IgG) Expected structural labelling (nuclear); validated and cited in human mouse and zebrafish (manufacturer's website)
TNNT2 (mouse IgG) Expected structural labelling (cytoskeleton); validated and cited in human and mouse (manufacturer's website)
ACTN2 (mouse IgG) Expected structural labelling (cytoskeleton); validated and cited in a very broad range of vertebrates including human and macaque (manufacturer's website)
MYL2 (rabbit IgG) Expected structural labelling (cytoskeleton); validated and cited in broad range of vertebrates (manufacturer's website)
TNNT2-FITC Validated in a broad range of mammals (manufacturer's website); validated on human cardiac cells with parallel negative non-cardiac controls (Miller et al. 2020, DOI: 10.1002/cpsc.125); analogous clone used on Macaca mulatta in Stauske et al. 2020, DOI: 10.3390/cells9061349)
MYL2-APC Validated in a broad range of mammals (manufacturer's website); validated on human cardiac cells with parallel negative non-cardiac controls (Miller et al. 2020, DOI: 10.1002/cpsc.125)

Eukaryotic cell lines

Policy information about cell lines and Sex and Gender in Research

Cell line source(s)

-Human induced pluripotent stem cells (iPSC) and iPSC-cardiomyocytes: A female human subject was recruited as part of a study at the Charite, Berlin, with broad consent given for human iPSC line generation and use for research purposes.
-Chimpanzee induced pluripotent stem cells (iPSC) and iPSC-cardiomyocytes: Generated from a male individual, source: Magdeburg zoo
-Gorilla induced pluripotent stem cells (iPSC) and iPSC-cardiomyocytes: Generated from a male individual, source: Rostock zoo
-Rhesus macaque induced pluripotent stem cells (iPSC) and iPSC-cardiomyocytes: Generated from a female individual, source: German Primate Center

Authentication

None of the cell lines used were authenticated. Gene markers were quantified and we did transcriptomic comparisons to other similar published cell lines in order to ensure that the cell lines correspond to each species iPSC-CMs

Mycoplasma contamination

Cell lines were tested for mycoplasma contamination as negative.

Commonly misidentified lines
(See ICLAC register)

Gene markers were quantified and we did transcriptomic comparisons to other similar published cell lines in order to ensure that the cell lines were not misidentified.

Animals and other research organisms

Policy information about studies involving animals; ARRIVE guidelines recommended for reporting animal research, and Sex and Gender in Research

Laboratory animals	N/A
Wild animals	Non-human primate left ventricles were retrived from the Biomedical Primate Research Centre (BPRC). The tissues from rhesus macaques were obtained from BPRC animals that were euthanized for welfare and ethical reasons. The chimpanzees' tissues were derived from animals residing at Safari Park Beekse Bergen and on which necropsies were done at the BPRC in the Netherlands.
Reporting on sex	Due to the existing limitations to collect non-human left ventricle primate data, we did not have enough samples representing both sexes for each of the species. Therefore, we did not consider sex in our study design, although both sexes were represented among non-human left ventricle replicates. Chimpanzee left ventricles were obtained for three males and one female. Rhesus macaque left ventricles were obtained for two males and two females.
Field-collected samples	N/A
Ethics oversight	BPRC complies with Article 4 (Principle of replacement, reduction, and refinement) and Article 47 (Alternative approaches) of the Directive 2010/63/EU on the Protection of Animals Used for Scientific Purposes. BPRC has been accredited by the Association for Assessment and Accreditation of Laboratory Animal Care (AAALAC) since 2012.

Note that full information on the approval of the study protocol must also be provided in the manuscript.

Plants

Seed stocks	N/A
Novel plant genotypes	N/A
Authentication	N/A

Flow Cytometry

Plots

Confirm that:

☒ The axis labels state the marker and fluorochrome used (e.g. CD4-FITC).

☒ The axis scales are clearly visible. Include numbers along axes only for bottom left plot of group (a 'group' is an analysis of identical markers).

☒ All plots are contour plots with outliers or pseudocolor plots.

☒ A numerical value for number of cells or percentage (with statistics) is provided.

Methodology

Sample preparation	Cells were harvested using 10x TrypLE (Thermo), stained for viability using VioBility Blue (Miltenyi), fixed and permeabilised using FoxP3 staining buffer kit (Miltenyi), and stained with conjugated antibodies.
Instrument	Expression was analysed using a MACSQuant VYB flow cytometer (Miltenyi) with gating.
Software	Plots were visualised using FlowJo 10.
Cell population abundance	A sample size of at least 10000 cells was included from the populations of whole single live cells, which represented >80% of their parent populations.
Gating strategy	Population gates were set for whole single live cells, with positive gates for expression targets set based on isotype antibody staining.

☒ Tick this box to confirm that a figure exemplifying the gating strategy is provided in the Supplementary Information.
